# Insights on Scan‐Specific Deep‐Learning Strategies for Brain MRI Parallel Imaging Reconstruction

**DOI:** 10.1002/nbm.70079

**Published:** 2025-06-22

**Authors:** Swetali Nimje, Thierry Artières, Maxime Guye, Ludovic de Rochefort

**Affiliations:** ^1^ Aix Marseille Univ CNRS, CRMBM, Institut Marseille Imaging Marseille France; ^2^ Laboratoire d'Informatique et Systèmes UMR 7020 Aix‐Marseille Univ., CNRS, Ecole Centrale de Marseille Marseille France; ^3^ APHM, Hôpital Universitaire Timone, CEMEREM Marseille France

**Keywords:** artifacts, Cartesian sampling, COBRAI, deep learning, parallel imaging, phase, reconstruction

## Abstract

Scan‐specific deep learning strategies have been proposed for parallel imaging reconstruction in which auto‐calibrated signals (ACS) are used for training. Here, we introduce methods to objectively optimize architecture and training details. In addition, we introduce a new metric to better characterize the quality of the reconstructed images. Various brain MRI situations are considered. The evaluated models encompass single‐layer and three‐layer residual CNN architectures with real and complex convolutions. Hyperparameters such as the level of linearity in leaky activation functions, loss function, kernel sizes and depths are optimized using grid‐search with K‐fold cross validation. The performances regarding ACS reference size and mode are also evaluated. An innovative COrrelation‐Based Residual Artifact Index (COBRAI) quantifying the level of structured residual artifacts is proposed. Qualitative and quantitative comparisons are performed both on the FastMRI and in‐house multi‐contrasts 2D data. The proposed objective grid‐search strategy based on ACS successfully provided optimized hyperparameters, retrospectively validated by enhanced image quality metrics. Notably, it is shown that nonlinearities produce structured residual artifacts, and that, among the models tested, a three‐layer residual linear CNN with complex implementation and a reduced number of parameters is more robust, particularly providing less structured artifacts with less training data, leading to larger acceleration rates. Deep‐learning MRI parallel image reconstruction in the scan‐specific approach can be optimized using grid‐search with K‐fold cross validation. It was successfully applied in various 2D brain MRI situations. The quantification of structured residual artifacts with COBRAI is a useful complementary characterization to state‐of‐the‐art metrics, and it can be used to drive model selection.

## Graphical abstract

Methods are proposed to optimize 2D brain MRI parallel imaging reconstruction using scan‐specific deep learning strategies. The study challenges the use of neural networks trained with limited ACS data and larger acceleration rates than clinical practice, notably proposing a new image metrics sensitive to structured residual artifacts to select models.

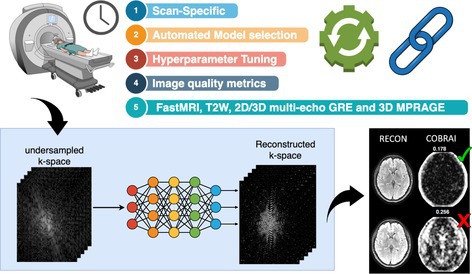

## 1 Introduction

Reducing MRI acquisition time is a constant goal to minimize motion artifacts, enhance patient comfort, and increase scanner availability, which can be achieved using Parallel Imaging (PI) for which a typical two to three acceleration rates are used in clinical practice. In recent years, deep learning techniques have been developed to solve MRI reconstruction tasks using various approaches, such as supervised learning (e.g., [1, 2, 3, 4, 5]), GAN‐based methods (e.g., [6, 7, 8, 9, 10, 11]), and self‐supervised methods (e.g., [12, 13, 14, 15]). While standard supervised learning can produce impressive results with higher acceleration rates and better image quality, it requires a large amount of training data and may not generalize well outside of the specific application on which it was trained. MRI is a complex method with a wide range of experimental conditions, such as multiple anatomic territories, manufacturer, user‐defined scan parameters, or a variety of sequence types, which can affect the accuracy of diagnosis if the training database does not include sufficient examples of all scan situations, especially pathologies of interest. Deep‐learning‐based MRI (DL‐MRI) models also do not generalize well across datasets collected with different coils [16], which is an important consideration for clinical adoption. To address these challenges, scan‐specific methods in which training data are acquired during the scan have been developed [15, 17, 18, 19, 20]. For example, robust artificial neural networks for k‐space interpolation (RAKI) [17] train neural networks on the AutoCalibration Signals (ACS) [21] to estimate unacquired lines in k‐space in a scan‐specific manner. For the latter strategy, there is a need to further investigate the performances in practical situations, which is the focus of this work.

Indeed, many prior studies based on the fastMRI dataset [22, 23] have focused on integrated‐mode ACS. In the integrated mode, the ACS is acquired along with the undersampled k‐space and shares the same contrast leading to a reduced effective acceleration rate. Alternatively, the ACS can be acquired in a separate mode prior to the undersampled k‐space. In contrast, the latter acquisition method incurs only a marginal increase in the total scan time because shorter repetition times can be used. The counterpart is that the magnitude and phase usually differ between the ACS and the undersampled k‐space, in particular, due to different echo times and acquisition bandwidths, such that scan‐specific models may fail to generalize well, leading to reduced image quality. This may be particularly true in applications in which the phase is of utmost importance for field mapping, phase‐contrast velocity mapping [24], fat‐water separation [25], or chemical shift imaging [26].

In addition, although complex convolution layers have been introduced in the DL community [27], many strategies, including RAKI, typically separate the real and imaginary components of complex numbers into separate real‐valued channels. Recent studies have shown that deep neural networks based on complex‐valued convolutions [28, 29] have a high level of representational power and accuracy and can be applied to MRI reconstruction, including both magnitude‐ and phase‐based applications. Thus, using real‐valued versus complex‐valued implementations may also have consequences for model generalizability and image quality. In this line, the recently proposed iRAKI [30] explored the use of complex‐valued networks in k‐space‐based reconstructions for 2D imaging. It is important to note that the optimization of various hyperparameters was performed empirically in these applications, for example, regarding the kernel sizes and depths, as well as the loss function. Additionally, the influence of the activation function, such as the traditional Rectified Linear Unit (ReLU) or its leaky extension that mitigates nonlinear effects, has not been evaluated.

Notably, the existing literature lacks comprehensive studies that investigate both optimal hyperparameters and model architecture for scan‐specific k‐space reconstruction tasks, including the influence of nonlinearities and real/complex implementations. While prior research has explored hyperparameter optimization in general machine‐learning contexts, their specific application to the domain of scan‐specific k‐space reconstruction remains limited. Additionally, reliable image quality evaluation is a major challenge in evaluating the quality of MRI reconstructions. Commonly reported metrics, such as Normalized Root Mean Square Error (NRMSE), Normalized Mean Absolute Error (NMAE), Peak Signal‐to‐Noise Ratio (PSNR), and Structural Similarity Index (SSIM), often fail to adequately account for local variations and fine details, leading to suboptimal assessments [31, 32], in particular, of visible residual artifacts.

To investigate these issues, methods are implemented in this work to objectively compare the performances. Our work aims to conduct a thorough investigation into the impact of hyperparameters on the reconstruction performance and to evaluate the generalizability with regard to the ACS mode and size. To achieve this, we employ a grid search strategy combined with K‐fold cross‐validation and systematically explore a range of hyperparameter values to identify optimal configurations that maximize the accuracy and effectiveness of the models and guide hyperparameter choice. As such, we leverage a train‐validation partition (TVP) scheme with early stopping to avoid or reduce overfitting effects. To address the issue of reconstruction quality evaluation, we also introduce the COrrelation‐Based Residual Artifact Index (COBRAI), a metric for image quality assessment sensitive to the presence of structured residuals in the image reconstructed from under‐sampled k‐space data as compared to ground‐truth image reconstructed from fully‐sampled k‐space data.

Focusing on implications for future research and clinical transfer, comparison of residual RAKI (rRAKI) [18], its complex counterpart (crRAKI) and GRAPPA [21] is performed in various brain imaging situations, using different ACS modes and sizes, in 2D with various contrasts (T1W, T2W, T2*W, FLAIR), as well as phase‐mapping with higher acceleration rates than the ones used in clinical practice.

## 2 Method

### 2.1 The Reconstruction Task

MRI Cartesian k‐space‐based PI can be summarized as the task of up‐sampling multicoil undersampled k‐space data. In this study, we focus on uniform undersampling and scan‐specific reconstruction, where the model is learned from the ACS and applied to every specific acquisition. In 2D, a *full k‐space*, $S_{f}$, is a 3D tensor of complex numbers, defined as

(1) $$S_{f}=\left\{S(i,j,c)\in C,i,j,c\in ⟦1,N_{x}⟧\times ⟦1,N_{y}⟧\times ⟦1,N_{c}⟧\right\}$$

where $N_{x}$ is the number of sampled points along the readout direction, $N_{y}$ is the number of sampled points in the phase encoding direction and $N_{c}$ is the number of coils. A single complex‐valued image I can be computed from the full k‐space tensor $S_{f}$ through Fourier transforms for each coil and by applying a coil combination algorithm such as *adaptive combination* [33, 34].

In the case of uniform undersampling that we consider here, the readout direction ($k_{x}$, the first dimension of the tensor S) is fully sampled while the phase encoding dimension ($k_{y}$, the second dimension of the tensor S) is undersampled. Assuming an acceleration rate equal to $R_{y}\in \mathbb{N}$ along $k_{y}$, an acquired *undersampled k‐space* may then be defined as the following 3D tensor:

(2) $$S_{u}=\left\{S(i,1+(j-1)\times R_{y},c)\in C,i,j,c\in ⟦1,N_{x}⟧\times ⟦1,\frac{N_{y}}{R_{y}}⟧\times ⟦1,N_{c}⟧\right\}$$

Acquiring such an *undersampled k‐space* (one line is acquired every $R_{y}$ lines) fastens the *full k‐space* acquisition time (which may typically last minutes) by a factor $R=R_{y}$ provided one is able to accurately reconstruct the unacquired lines.

Generally speaking, a reconstruction model operating in k‐space takes an *undersampled k‐space* tensor $S_{u}$ as input and outputs its *full k‐space* approximation ${\hat{S}}_{f}$:

(3) $${\hat{S}}_{f}=f_{\theta }(S_{u})$$

Approximating $S_{f}$, that is, computing ${\hat{S}}_{f}$, can be decomposed into (R−1) (independent) subtasks, where each subtask corresponds to the approximation of an undersampled view of $S_{f}$. Let decompose the full k‐space in a series of $R_{y}$ subsets of the full k‐space, that is, undersampled k‐spaces. Let note $S_{u}(m)$ (where $m\in \left[\;\left[1,R_{y}\right]\;\right]$), the $m^{th}$ *undersampled k‐space* tensor below:

(4) $$S_{u}(m)=\left\{S(i,m+(j-1)\times R_{y},c)\in C,i,j,c\in ⟦1,N_{x}⟧\times ⟦1,\frac{N_{y}}{R_{y}}⟧\times ⟦1,N_{c}⟧\right\}$$

Then the full k‐space is the union of all these $R_{y}$ undersampled views of the full k‐space, $S_{f}=\{S_{u}(1),\ldots S_{u}(R_{y})\}$. Assume that $S_{u}(1)$ has been acquired. The goal of a reconstruction model, f, is to predict $\left\{{\hat{S}}_{f}(2),\ldots {\hat{S}}_{f}(R_{y})\right\}$ which are estimations of the $\left(R_{y}-1\right)$ unacquired undersampled k‐space views which, together with the acquired $S_{u}(1)$ forms an estimate of the full k‐space ${\hat{S}}_{f}=\{S_{u}(1),{\hat{S}}_{f}(2),\ldots {\hat{S}}_{f}(R_{y})\}$ from which an approximated image may be reconstructed.

In our approach, each of the unacquired *undersampled k‐spaces* (${\hat{S}}_{f}(m)$) is estimated using a specific reconstruction model $f_{\theta_{m}}$ from the acquired *undersampled k‐space*, according to

(5) $${\hat{S}}_{f}(m)=f_{\theta_{m}}(S_{u})$$

As said previously the *full k‐space* reconstruction ${\hat{S}}_{f}$ is achieved by combining the $\left(R_{y}-1\right)$ estimated *undersampled k‐spaces* ${\left({\hat{S}}_{f}(m)\right)}_{m}$ obtained using the reconstruction models and the acquired undersample k‐space $S_{u}$. We choose to use the same architecture for each model $f_{\theta_{m}}$ and train each independently.

The ACS that will be used for training the model correspond to a fully‐sampled *small* k‐space, that is, a 3D tensor of complex numbers:

(6) $$S_{f}^{acs}=\left\{S^{acs}(i,j,c)\in C,i,j,c\in ⟦1,N_{x}^{acs}⟧\times ⟦1,N_{y}^{acs}⟧\times ⟦1,N_{c}⟧\right\}$$

where $N_{x}^{acs}\leq N_{x}$ is the number of sampled points along the readout direction for the ACS and $N_{y}^{acs}\leq N_{y}$ is the number of sampled points in the first phase encoding direction. These ACS are typically acquired at the same location and with the same field‐of‐view, resulting in the same k‐space distance between the sampled points. However, they can be acquired with the same sequence timings (integrated mode), or different timings (separated mode), leading to the same or different underlying image contrasts, respectively. In integrated mode, the ACS lines are consequently integrated to the undersampled k‐space and needs not to be estimated, leading to a smaller effective acceleration rate, as will be discussed later in the manuscript.

### 2.2 Training and Validation Strategies With ACS Partition

**FIGURE 1:**
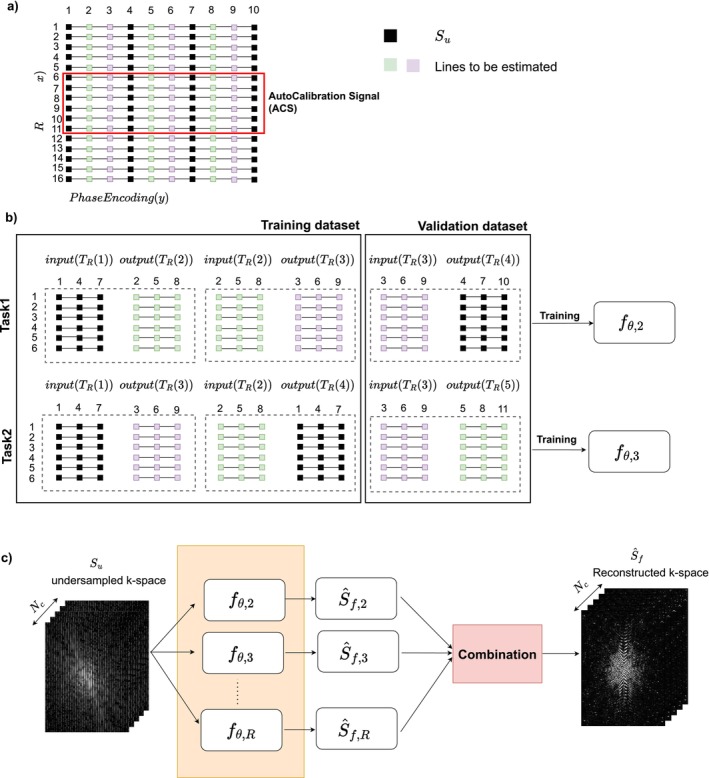
Illustration of training strategy in 2D for an acceleration factor $R_{y}=3$. (a) The acquired *undersampled k‐space* includes lines numbered 1, 4, 7, … while lines 2, 3, 5, 6, 8, 9, … are unobserved. The learning is performed on a small part of the*k‐space* (ACS), which is fully acquired. (b) The fully acquired ACS is artificially undersampled to build supervised datasets for learning reconstruction models. There are R−1 reconstruction tasks for an acceleration factor R, and one reconstruction model is learnt on each one. To select the best model architecture one uses a train/validation split. (c) After the training is performed for each task, the estimations of each model on a new *undersampled k‐space* are combined into an estimated *full k‐space*.

We focused on the PI reconstruction task of estimating a fully‐sampled multi‐coil k‐space that has been uniformly undersampled as illustrated in Figure 1 and detailed in Section 2.1. A model $f_{\theta }$ takes as input observed under‐sampled multi‐coil k‐space data and infers a full multi‐coil k‐space. In our implementation, the model is divided into several models trained separately, each taking the same observed under‐sampled multi‐coil k‐space data as input, but inferring a specific missing multi‐coil k‐space line. The models are trained on ACS data that are retrospectively undersampled and used to infer the target missing k‐space line. It is common practice in deep‐learning to split the reference data into training and validation sets in order to evaluate the performance of the model during training. As there are R sets of pairs (input and output lines), we distinguish between training data and validation data, which are not used to learn the model but to estimate its prediction ability and prevent overfitting. Therefore, we used (R−1) sets of pairs to learn the model and the last set for validation. We refer to this method as Train‐Validation Partition (TVP).

Figure 1a illustrates how this split is done, that is, how a fully sampled multi‐coil k‐space (the ACS in our case) is used to learn a reconstruction model for an acceleration rate along the phase encoding direction $R_{y}=3$. We assume that the acquired lines in the phase‐encoding direction are indexed by $T_{R}(1)\equiv \{1,4,7,\ldots ,\}$ (see Figure 1a). The task is to build models capable of inferring lines using the indices in $T_{R}(2)\equiv \{2,5,8,...\}$ and $T_{R}(3)\equiv \{3,6,9,\ldots ,\}$. Clearly, the reconstructed lines in $T_{R}(2)$ from those in $T_{R}(1)$ are the same as the reconstructed lines in $T_{R}(3)$ from those in $T_{R}(2)$, but the reconstructed lines in $T_{R}(3)$ from those in $T_{R}(1)$ are different. Thus, (R−1) reconstruction subtasks exist, and each learns a distinct reconstruction model. For example, the first model is trained to reconstruct the first missing lines from observed lines (Task 1). It is trained to infer lines in $T_{R}(2)$ from $T_{R}(1)$ and infer lines in $T_{R}(3)$ from lines in $T_{R}(2)$. For example, for Task 1 in Figure 1a, the last pair of input ($T_{R}(3)$) and output lines ($T_{R}(4)$) are reserved for validation.

**FIGURE 2:**
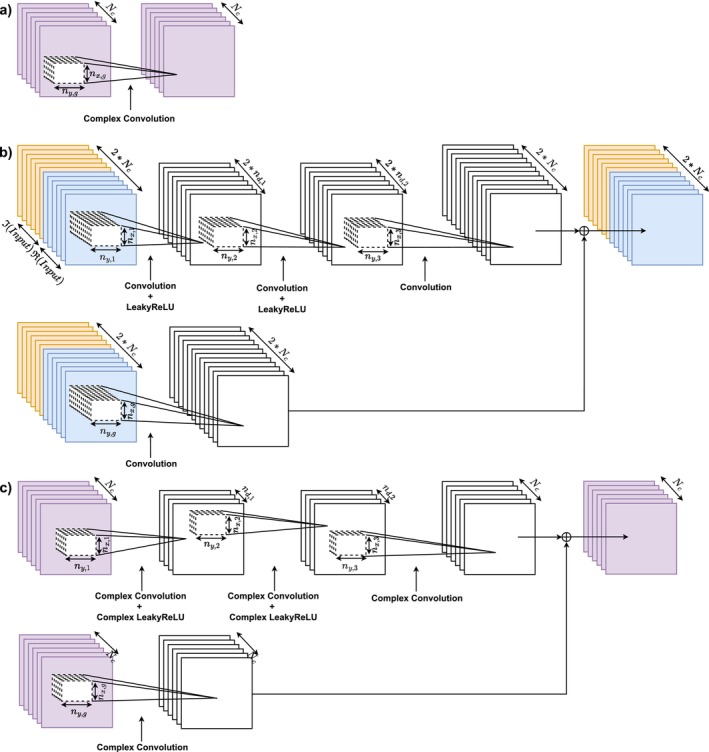
Network architectures for 2D reconstruction: (a) GRAPPA, (b) rRAKI architecture with its real‐valued implementation concatenating the real and imaginary parts, and (c) crRAKI (shown here for the 2D case). Note that real‐valued implementation of convolutional layers (e.g., rRAKI) include twice more maps than complex‐valued implementation (crRAKI) since real and imaginary parts are separately computed in different real maps. Both rRAKI and crRAKI use two paths to process a single input multi‐coil *undersampled k‐space*, which is drawn twice here to improve clarity, a GRAPPA short connection and a RAKI/cRAKI section, whose outputs are summed to produce the predicted output.

### 2.3 GRAPPA, Residual RAKI, and Complex Residual RAKI Models

GRAPPA [21] is a linear model that estimates a missing value at a position in a grid by using a combination of values in neighboring locations. However, in the DL‐MRI context, the GRAPPA model network architecture is a single‐layer neural network with complex‐valued convolutional and without bias (see Figure 2). Its architecture is defined by the size of the convolution filter, $\left(n_{x,g},n_{y,g}\right)$. While there are several ways to determine the GRAPPA kernel weights, a regularized strategy is often required when the ACS size is small compared to the number of kernel weights. This regularization is implemented here in two ways: an iterative DL‐MRI framework using TVP and the MSE loss, as well as with Tikhonov regularization [35] which are, respectively, referred to as GRAPPA(TVP) and GRAPPA(TR). The following kernel sizes are considered for optimization in this work: [$n_{x,g},\;n_{y,g}$] = [3, 2], [5, 2], [5, 4], [5, 5], [7, 2], [7, 4], [9, 2], [9, 4].

The rRAKI model [18] implements a linear residual connection part (referred to as a GRAPPA short connection) together with a three‐layer CNN (RAKI) section [36] (see Figure 2 ). We use the line‐by‐line implementation [37] here, using real (rRAKI) and complex (crRAKI) implementations of convolutions and activation functions. LeakyReLU was used as it has been reported to improve stability [38] and can be used to scale the amount of nonlinearity (the negative slope is noted c). This architecture is defined by several hyperparameters: the size of the convolutional filters of the first layer $\left(n_{x,1},n_{y,1}\right)$ and of the second $\left(n_{x,2},n_{y,2}\right)$ and third layers $\left(n_{x,3},n_{y,3}\right)$, the depth in the two first convolutional layers ($n_{d,1},\;n_{d,2}$). Different combinations are considered for optimization (72 in total): first layer size [$n_{x,1},\;n_{y,1}$] = [5, 2] and [5, 4]; second layer size set to [$n_{x,2},\;n_{y,2}$] = [1, 1]; layer depths $2\times n_{d,1}$ and $2\times n_{d,2}$ = 32, 16, and 8; third layer size [$n_{x,3},\;n_{y,3}$] = [3, 2] and [3, 4]; residual layer size [$n_{x,g},\;n_{y,g}$] = [5, 2] and [1, 1].

### 2.4 Data

Reconstruction was performed on the fastMRI dataset that encompasses fully‐sampled k‐space enabling to simulate integrated‐mode ACS [23] acquisitions, as well as in‐house separated‐mode ACS scans. For the latter, 3T (Vida, Siemens, Erlangen, Germany) brain raw data were used, and acquisitions consisted in brain scans using T2 W and 2D multi‐echo gradient echo (GRE) sequences.

#### 2.4.1 FastMRI Data

Twenty volumes were randomly selected for each of the four different brain contrasts available, namely, FLAIR, T1W, T2W, and T1POST, resulting in approximately 300 slices for each contrast from the fastMRI dataset. To simulate undersampled acquisitions, full k‐spaces were retrospectively undersampled for various $R_{y}$ keeping 24 and 40 ACS lines.

#### 2.4.2 T2‐Weighted Spin‐Echo

Interleaved multi‐slice axial T2 W dual‐echo ($TE_{1}$/$TE_{2}$ = 11/90 ms) 2D spin‐echo brain images were acquired with a 64‐channel head coil ($N_{c}$ = 52 selected). Field‐of‐View (FOV) was 250 × 187.5 mm and the slice thickness was 3 mm. Repetition and inversion times were respectively 9020 and 900 ms, and the flip angle was 150°. Fully‐sampled k‐space (total acquisition time $T_{full}$ = 4.1 min), as well as undersampled ones with $R_{y}$ = 2–6 were acquired. ACS were acquired in separated mode (40 ACS acquired in $T_{ACS}$ = 8.2 s).

#### 2.4.3 2D Multi‐Echo GRE

A first setup was used to acquire interleaved multi‐slice (24 slices) 2D GRE multi‐echo (6 echos, TE1/dTE = 3.2/5.23 ms) transverse brain acquisitions with FOV = 256 × 256 mm, slice thickness 3 mm, 80% phase encoding resolution, TR = 802 ms, $T_{full}$ = 2.4 min, flip angle = 70°, with a 20‐channel coil array ($N_{c}$ = 16 selected). Acquisitions with acceleration factors $R_{y}$ = 2 were performed with 24 ACS lines in separated mode. To study the effect of ACS size, a second setup was used to acquire a fully sampled 2D GRE multi‐echo (6 echoes) with a 64‐channel coil array ($N_{c}$ = 52 selected). ACS were also acquired in separated mode (64 ACS acquired in $T_{ACS}$ = 5 s).

We later report the effective acceleration rates in the different simulated situations. In integrated mode, the effective acceleration rate can be estimated as $R_{eff}=R_{y}\times N_{y}/(N_{y}+(R_{y}-1)\times N_{y}^{acs})$, while in separated mode $R_{eff}=(T_{ACS}+T_{full})/(T_{ACS}+T_{full}/R_{y})$.

### 2.5 Training Implementation

The study employed Python as the software platform for development and Pytorch as the core tool for building and training the model. To ensure efficient computation, the training process was performed on a GPU‐powered slurm cluster equipped with an NVIDIA GeForce RTX 2080 Ti GPU. It was also running on personal laptop computers when limited number of slices were to be reconstructed. The reference ACS data were first normalized by the 2‐norm of all samples and coils. PyTorch default weight initialization is used where the weights of a layer are random values drawn from a normal distribution with zero mean and standard deviation of $\sqrt{\frac{2}{n_{\text{in}}}}$, where $n_{\text{in}}$ is the number of input units to the layer. A maximum of 1000 training iterations (epochs) were conducted using the ADAM optimizer and a learning rate of 10^−3^. The early‐stopping strategy to prevent overfitting consists in evaluating the model's performance on the validation set at each epoch. The training was terminated when the performance on the validation set began to deteriorate, specifically when the MSE loss on the validation set increased for more than 20 consecutive epochs. The model parameters corresponding to the epoch with the lowest MSE loss on the validation set were retained for evaluation.

### 2.6 Grid‐Search With K‐Fold Cross‐Validation to Select Hyperparameters

A comprehensive grid‐search was conducted in combination with K‐fold cross‐validation, which entails dividing the available data into K equal number of subsets, or “folds,” and training and evaluating the model on each subset. This method offers a more robust evaluation of a model's performance, particularly its ability to generalize to new data. Specifically, in the context of the k‐space reconstruction task, K was set equal to R to reflect the alignment with the number of folds derived from the R datasets discussed in Section 2.2. In each round of training and validation, one of the R folds was used as the validation set, whereas the remaining R−1 folds were used as the training set. The model was trained on the training set and its performance was evaluated on the validation set. This process was repeated R times, and each fold was used once as the validation set. The results from each round of validation were averaged to obtain an overall estimate of the model's performance. The evaluation metric for model performance was the Mean Squared Error (MSE) in k‐space for the validation set. By averaging the results from each round of validation, we obtained a more accurate representation of the model's overall performance and its potential for generalization.

The same loss function as in the original paper [18] is used here for the training of rRAKI and crRAKI models (see eq. 6 in the reference). The GRAPPA short connection and RAKI/cRAKI sections are explicitly separated and trained jointly. Training is performed via a weighted MSE loss function consisting of two terms: the first term calculates the loss between the rRAKI/cRAKI prediction and the actual output (model loss), and the second term consists of the loss between the GRAPPA short‐connection prediction and the actual output (short‐connection loss). A weighting factor, λ, is introduced in front of the second term to define the total loss function. In the following, we refer the extreme cases to as linear (L) rRAKI/crRAKI with the parameters λ=0 and c=1 and to nonlinear (NL) counterparts with λ=1 and c=0.

#### 2.6.1 Kernel Sizes and Depths Optimization

GRAPPA(TVP), rRAKi(NL), and crRAKI(L) models can be challenged on their kernel sizes and depths by training these models for all the combinations listed in Section 2.3 and for $R_{y}$ = 2–6. When prospectively evaluating the performance based on the ACS only (before the trained model is applied to new data), the method with the lowest mean k‐space MSE over all slices for the validation set is determined and considered the best for a given acceleration rate. A one‐tail Student's t‐test assuming unequal variance is then performed to test the hypothesis that this architecture is significantly producing a smaller k‐space MSE on average than the other architecture kernel sizes and depths. The architecture kernel sizes and depths for which this hypothesis can be rejected (when p<0.05) are then considered optimal kernel parameters that can be chosen for this acceleration rate. Finally, kernel sizes and depths can be selected based on its efficiency for several acceleration rates and, where appropriate, based on its total number of trainable parameters.

#### 2.6.2 Loss Function and Linearity

Grid‐search with K‐fold cross validation is used to evaluate the impact of adding the mixed loss and to find the optimal value for c. For this experiment, multi‐slice T2 W spin‐echo ACS data is used, with $R_{y}$ = 5 and $N_{y}^{acs}$ = 40, together with the architecture kernel and depth sizes used in the original rRAKI architecture. The tested linearity parameters are $c\in \left[\;\left[0,0.01,0.1,0.2,0.3,0.5,0.7,0.9,1\right]\;\right]$, and $\lambda \in \left[\;\left[0,1\right]\;\right]$ is used to exclude/include the short‐connection loss in the total loss (referred to as the ablation study). The value of c that produced the smallest MSE on average for all slices was selected as the optimal choice, and, to validate the significance of the optimal c value, one‐tailed Student's *t*‐tests were performed to compare its performance with the models trained with other values of c.

### 2.7 Retrospective Image Quality Metrics: Focus on Structured Residual Artifacts

After training has been performed using the ACS, the model is applied to new under‐sampled k‐space data that can be used to predict the unacquired k‐space data and ultimately compute an approximated reconstructed image. The later is obtained after a Fourier transform and a coil combination (using either sum‐of‐squares. Additionally, complex images from the first echo were low‐pass filtered and used as the estimated complex coil sensitivity for a phase‐preserving coil combination. Ground‐truth images are reconstructed similarly from fully‐acquired k‐spaces in order to evaluate several commonly used metrics [4]. The NRMSE (Normalized Root Mean Square Error) and PSNR (peak signal‐to‐noise ratio) were used to compare the intensity differences between the reconstructed and reference images at the pixel level and to assess the visibility of the signal in the image compared to the noise. We also computed NMAE (Normalized Mean Absolute Error) and the SSIM (structural similarity index) as evaluation criteria as well as the Blur metric [39].

**FIGURE 3:**
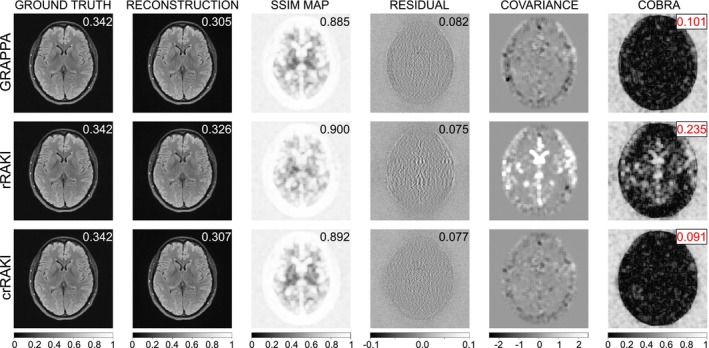
Illustration of the metrics to compare GRAPPA (first line), rRAKI (second line), and crRAKI (third line) reconstructions (FLAIR fastmri acquisition id 200_6002425, slice index 0 with $N_{y}^{acs}$ = 40, $R_{y}$ = 4 and $R_{eff}$ = 2.91). First column: normalized ground truth coil‐combined magnitude image. Second column: normalized reconstructed magnitude image. Third column: SSIM Map. Fourth column: residual maps. Fifth column: patch‐based covariance between the ground‐truth and the residual maps. Sixth column: COrrelation‐Based Residual Artifact maps (COBRA, that is, the local correlation coefficient). The number in the image top‐right corner is the mean over a mask encompassing the brain of the blur metric (for ground truth and reconstructed images), of the mean SSIM (for the SSIM map), the mean RMSE (for the residual map), and COBRAI. While rRAKI is slightly better in terms of Blur metrics, SSIM and RMSE, the covariance and COBRA map highlights the presence of structured residual artifacts, which is synthesized by a larger COBRAI. In this example, GRAPPA and crRAKI have smaller COBRAI values, that is, fewer structured artifacts.

Together with these standard image quality metrics, to assess the presence of residual artifacts and compare magnitude image reconstruction quality, COBRAI is proposed as an average local measure of residual artifacts. Similar to SSIM, COBRAI utilizes a patch‐based approach. For each patch in an image (chosen here as an 11 × 11 pixels kernel as in SSIM default computation), the correlation coefficient between Residual Maps (RM) of the predicted reconstruction and the ground truth is computed. The COBRAI metric is then taken as the mean absolute value of the correlation coefficients of all patches within a relevant brain mask. This process is illustrated in Figure 3 on which structural artifacts in the residual map become apparent. The resulting COBRAI scores ranged from 0 to 1. A perfect reconstruction is expected to lead to unstructured noise in the RM when the reconstruction captures the details accurately, thus having a low correlation with the ground truth. Thus, when comparing several reconstructions with COBRAI, a lower score indicates a lower average correlation between local patches in the corresponding RM as compared to the ground truth. At the same time, a higher COBRAI implies that the corresponding RM has more structural information which is either not present in the ground truth (hallucinations), or that details that are present in the ground truth are not reflected in the current reconstruction. To evaluate the performance retrospectively in terms of image metrics, the method providing the best results on average was compared to the others using a one‐tail paired Student's *t*‐test (consistently with earlier work [18]) after a normality test (Shapiro–Wilk). For all tests, alpha‐risks of 0.05 were considered significant.

## 3 Results

### 3.1 Grid‐Search With K‐Fold Cross‐Validation to Select Hyperparameters

#### 3.1.1 Kernel Sizes and Depths Optimization

The grid search yielded a list of architecture sizes and depths for GRAPPA, rRAKI, and crRAKI (Supporting Information Table S1) prone to produce minimal MSE in k‐space for several acceleration rates. Interestingly, GRAPPA and rRAKI sizes selected in earlier RAKI‐based works [17, 18] were found in this list and consequently selected for the next experiments (Table 1). In particular, for crRAKI, the residual layer size did not significantly influence the performance in terms of k‐space MSE; therefore, the smallest size was selected for this layer to reduce the total number of model parameters.

**TABLE 1:** Model hyperparameter search result.

Model	Structure	[$n_{x,l},n_{y,l},n_{d}^{in},\;n_{d}^{out}$]
GRAPPA (complex)	Single layer	[5, 4, $N_{c},\;N_{c}$] ×2
rRAKI (real)	Layer 1	[5, 2, $2\times N_{c}$, 32]
	Layer 2	[1, 1, 32, 8]
	Layer 3	[3, 2, 8, $2\times N_{c}$]
	residual layer	[5, 2, $2\times N_{c},\;2\times N_{c}$]
crRAKI (complex)	Layer 1	[5, 2, $N_{c}$, 16] ×2
	Layer 2	[1, 1, 16, 32] ×2
	Layer 3	[3, 2, 32, $N_{c}$] ×2
	Residual layer	[1, 1, $N_{c},\;N_{c}$] ×2

*Note:* Kernel sizes reported for each layer and model. $N_{c}$ is the number of coils. $n_{d}^{in}$ and $n_{d}^{out}$ stand for input and output depth, respectively. Two kernels (×2) are used for complex convolution.

#### 3.1.2 Loss Function and Linearity

**FIGURE 4:**
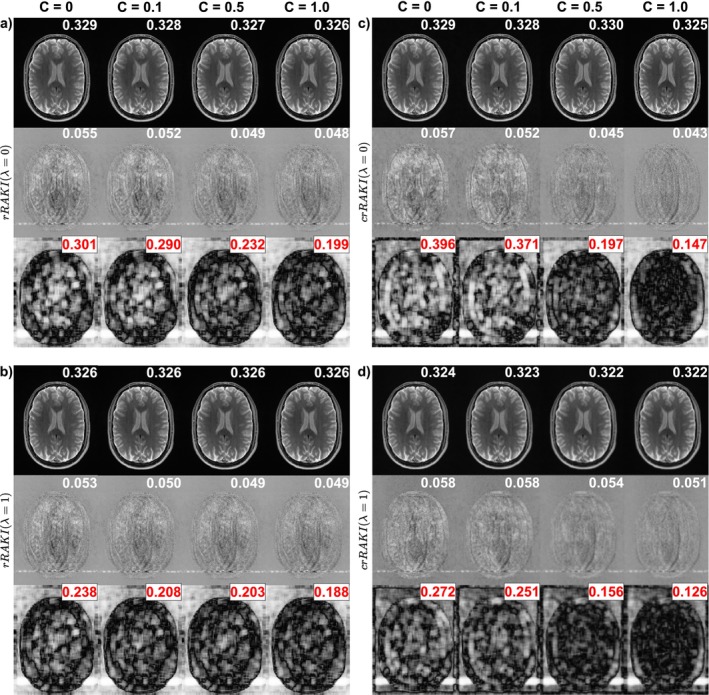
Illustration of the effects of nonlinearities for $R_{y}$ = 4 on the T2 W Spin‐Echo data with $N_{y}^{acs}$ = 40 ($R_{eff}$ = 3.65) using rRAKI (a, b) and crRAKI (c, d) with LeakyReLU activation function by varying the coefficient parameter c. The top set corresponds to λ=0 (a, c) and the bottom set corresponds to adding a short‐connection loss with λ=1 (b, d). For each set, the first row if the reconstructed magnitude image, the second row is the residual, the third row is the COBRA map. The numbers on the top‐right corners are respectively the Blur metrics, the mean RMSE, the COBRAI taken over a brain mask. As can be seen, both structured artifacts decrease with increasing c, associated with a reduction of RMSE and COBRAI. The addition of a short‐connection loss enhances the results, enforcing linearity of this section of the models. Best results are obtained when fully linear models are used, regardless the addition of a short‐connection loss.

The ablation study (Figure 4 illustrating $R_{y}$ = 4, and Supporting Information Figure S1 illustrating $R_{y}$ = 5, Tables S2 and S3) indicates that, when nonlinearities are used, the short‐connection loss plays a significant role in the overall performance of rRAKI and crRAKI, respectively, as removing this loss resulted in a notable decrease in the reconstruction performance and visual quality, particularly for c=0 (pure ReLU). The qualitative performance was confirmed by the quantitative performance evaluated using KSPACE‐MSE from k‐fold cross‐validation, NRMSE, NMAE, SSIM, Blur metrics, and COBRAI. The KSPACE‐MSE, NRMSE, and NMAE metrics demonstrated the best performance when rRAKI was linear, exhibiting superior reconstruction quality. Notably, the KSPACE‐MSE was minimal for λ=0 and c=1 for both rRAKI and crRAKI, without a significant difference compared to λ=1 and c=1 for rRAKI. These equivalent optimal conditions also exhibited a smaller COBRAI. Consequently, the KSPACE‐MSE obtained from K‐fold cross‐validation can be used for hyperparameter selection, the linear variants of rRAKI/crRAKI performed better, and the short‐connection loss is needed only when the model is nonlinear.

### 3.2 Focus on Image Quality

#### 3.2.1 Retrospective Under‐Sampling: Linearity, ACS Size, and Mode

Overall, the results obtained with the TVP implementation are consistent with earlier studies comparing the performances of rRAKI and GRAPPA(TVP) with integrated ACS 40 in most cases and for all reported metrics that were used [18]. This provides confidence in our TVP scheme with an early stopping and reconstruction implementation. GRAPPA(TVP) sometimes outperforms GRAPPA(TR) with Tikhonov regularization in the case of FLAIR and T1W.

**FIGURE 5:**
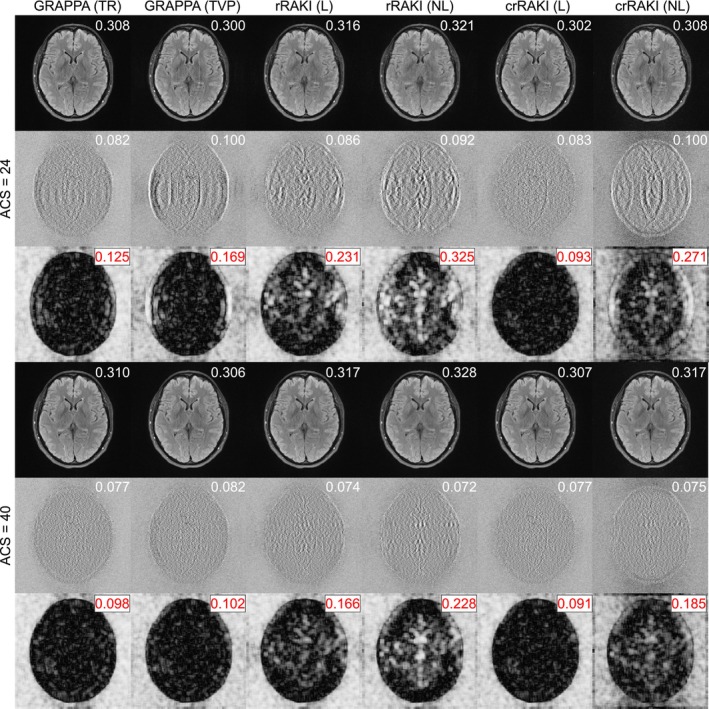
Comparison of the reconstruction for FastMRI FLAIR dataset id 200_6002425 slice index 0 for $R_{y}$ = 4 and two ACS sizes (24 and 40, $R_{eff}$ = 3.27 and 2.91, respectively). For each ACS set, the first row if the reconstructed magnitude image, the second row is the residual, the third row is the COBRA map. The numbers on the top‐right corners are respectively the Blur metrics, the mean RMSE, the COBRAI taken over a brain mask. GRAPPA with Tikhonov Regularization (TR) and Train‐Validation Partition (TVP), as well as rRAKI linear (L) and nonlinear (NL) perform well only when ACS size is large enough. rRAKI (NL) for $N_{y}^{acs}$ = 40 appears to be the one with lower NRMSE, but with significant structured residual artifacts. The crRAKI Linear method demonstrates superior capability in reconstructing high‐quality images with enhanced fidelity and accuracy.

Figure 5 provides a qualitative evaluation for a FLAIR image (see Supporting Information Figures S2, S3, and S4 for similar results on T1, T1POST, and T2 images). Specifically, crRAKI(L) exhibited superior visual quality compared to GRAPPA(TR) and GRAPPA(TVP), crRAKI(NL), and rRAKI(L/NL), while being less sensitivity to ACS size. It was characterized by smaller residuals and less structured artifacts. To illustrate the utility of COBRAI, in Figure 5, rRAKI(NL) has a lower NRMSE for $N_{y}^{ACS}=40$, and would be considered superior based on this criterion. However, the residual is more structured than, for example, crRAKI(L), which is captured by COBRAI, such that it is considered less efficient based on this metric. Supporting Information Tables S4 and S5 confirm these trends quantitatively in a larger number of reconstructed slices (300 per contrast). For ACS = 40, rRAKI demonstrated statistically significant improvements over GRAPPA and crRAKI in FLAIR and T1 W based on NRMSE, NMAE, SSIM and PSNR. However, overall, COBRAI was consistently lower for crRAKI(L) than for the other models across different ACS levels and contrasts, indicating lower structured artifact levels in the fastMRI dataset, in line with the trends observed in Section 3.1. This suggests that the standard metrics are not ideal to compare the model performances.

In order to compare integrated and separated mode scan‐specific reconstructions, the in‐house T2 W data was reconstructed in both modes. The separated mode leads to a larger effective acceleration rate than the integrated mode. Indeed, the former uses a separated acquisition with a different contrast (short TR and TE), while the later uses the same sequence parameters, and needs not to estimate the ACS locations. As can be seen (Supporting Information Figure S5), crRAKI provides lower NRMSE and lower COBRAI in both cases, with an expected reduced NRMSE in the integrated case.

#### 3.2.2 T2‐Weighted: Acceleration Rate

**FIGURE 6:**
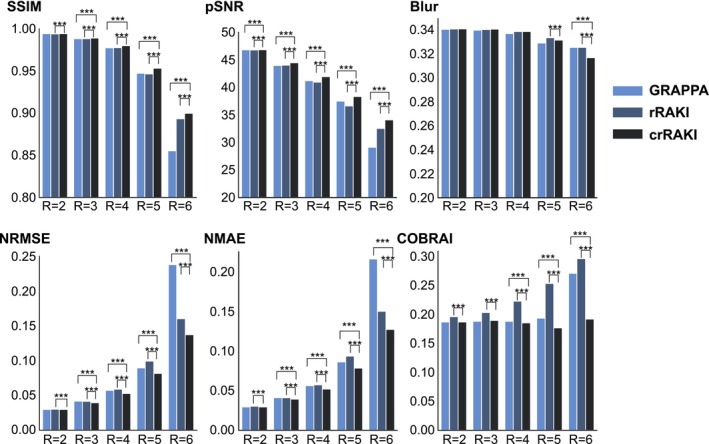
Quantitative evaluation of the reconstruction performance of GRAPPA, rRAKI, and crRAKI in terms of SSIM, PSNR, NRMSE, NMAE, Blur and COBRAI at acceleration rates of R = 2, 3, 4, 5, and 6 ($N_{y}^{acs}$ = 40, $R_{eff}$ = 1.94, 2.82, 3.65, 4.43, and 5.17). The values are the mean metric over all 49 slices for the two echoes of data from the T2 W scan on one volunteer, using the sum‐of‐squares coil combination. Accompanying p‐values (*, **, ***) calculated from a one‐tailed paired t‐test are displayed. A small p‐value (typically less than 0.05) indicates strong evidence against the null hypothesis (that the methods have equal performance) and supports the alternative hypothesis (that crRAKI has better performance than the other methods) The symbols *, **, and *** in statistical t‐tests are used to indicate the level of statistical significance of the results. * signifies p<0.05 (5% level), ** signifies p<0.01 (1% level), and *** signifies p<0.001 (0.1% level).

Figure 6 shows a comparison of the quantitative performance for different acceleration rates. The comparison was performed under the same experimental conditions in terms of the number of ACS lines as suggested by Zhang et al. [18]. The statistical results showed that crRAKI outperformed GRAPPA and rRAKI in all metrics except blur metrics at $R_{y}$ = 3, 4, 5, 6, the exception being GRAPPA at $R_{y}$ = 2. crRAKI demonstrates significant superiority over both rRAKI and GRAPPA in terms of NRMSE, NMAE, PSNR, and COBRAI, particularly at acceleration rates of 4, 5, and 6.

We observed that for $R_{y}$ = 2, 3, both rRAKI and crRAKI were able to reconstruct images that matched GRAPPA reconstruction quality. At $R_{y}$ = 4, 5, we start to see undersampling artifacts in the GRAPPA and rRAKI reconstructions, which are indicated by the residual maps shown in Supporting Information Figure S6 . In contrast, the crRAKI method has fewer artifacts, suggesting that it can better preserve the global features of the images. At $R_{y}$ = 6, the GRAPPA method significantly degraded the reconstruction, resulting in images of poor quality and low fidelity. The rRAKI method produces noisy reconstructions, which makes it difficult to see the details of the images. Overall, the crRAKI method produced reconstructions with an acceptable visual quality of up to $R_{y}$ = 5. Even in noisier reconstructions at $R_{y}$ = 6, the overall features of the image are preserved, making the images produced by this method visually acceptable, even at this high value of R.

#### 3.2.3 2D Multi‐Echo GRE: Phase Map Evaluation

**FIGURE 7:**
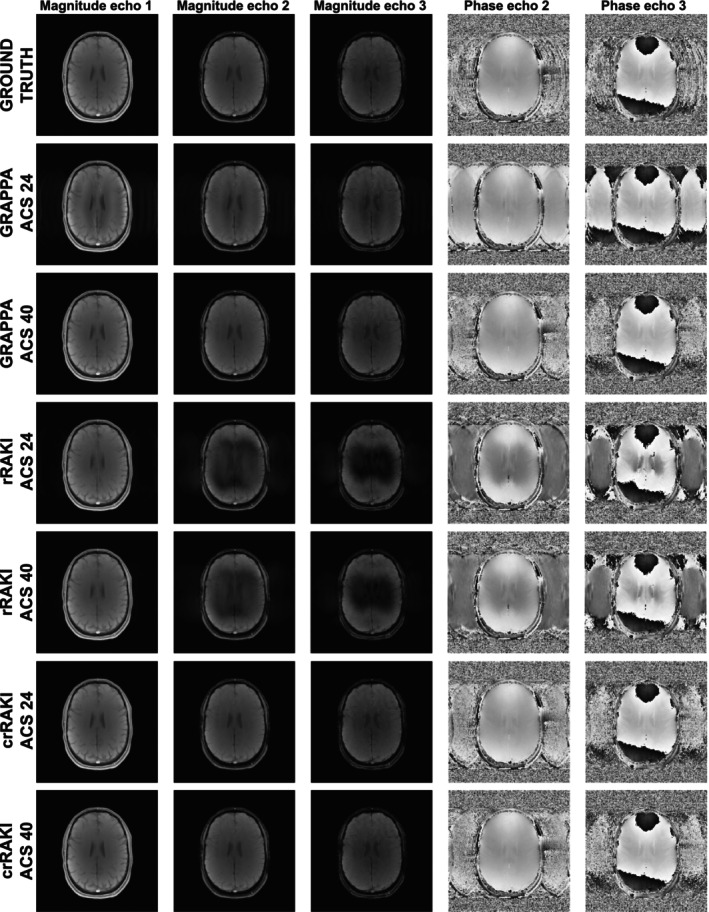
Magnitude and phase 2D GRE reconstructions with $R_{y}$ = 4 for 3 echoes for GRAPPA, rRAKI, and crRAKI with 24 and 40 ACS lines ($R_{eff}$ = 3.85 and 3.76). The first row corresponds to the ground‐truth fully‐acquired images, on which limited motion‐induced phase artifacts can be seen in the left‐right direction. For $N_{y}^{acs}$ = 24, GRAPPA displays residual undersampling artifacts both in magnitude and phase images (the brain is reproduced every fourth field‐of‐view in the left‐right direction) that disappear for $N_{y}^{acs}$ = 40, indicating that the number of ACS is adequate to learn the model parameters. Regardless of the number of ACS, rRAKI magnitude images display signal voids in the central part of the brain and phase images have remaining undersampling artifacts, with an increase with echo time. crRAKI‐generated images do not display these artifacts and are closer to the ground‐truth. Notably, the reduced sensitivity of crRAKI to the ACS size as compared to GRAPPA is expected given its reduced number of parameters.

Figure 7 compares the performances of the three different methods in a 2D multi‐echo GRE acquisition for $N_{y}^{acs}$ = 24 and 40 on magnitude and phase images. The results show that the GRAPPA performances highly depend on the number of ACS, and that rRAKI produces strong undersampling artifacts. In contrast, the crRAKI method has almost no undersampling artifacts in either the magnitude or phase images at $R_{y}$ = 4 for the two tested ACS cases. This indicates that the crRAKI method is capable of performing more accurate phase reconstructions at higher values of R, such as $R_{y}$ = 4, in these 2D acquisition settings. It is important to note that there exists a difference in contrast information between the ACS and the undersampled image scan. A noticeable degradation was observed in the rRAKI reconstruction as echo time increases, indicating a limited generalization capability that most probably result from learning the specific ACS phase which is acquired for a small echo‐time, thus not adapted to increasing echo times.

## 4 Discussion

### 4.1 Main Findings

This study focused on optimizing scan‐specific reconstruction models in 2D, comparing GRAPPA, rRAKI, and its complex counter‐part crRAKI. We have shown that grid‐search using ACS only can be used to select objectively kernel sizes and depths, as well as optimize the loss function and the amount of nonlinearity in activation functions, elements that were not evaluated in earlier works. For acceleration rates higher than current clinical practice, image quality was then evaluated both on FastMRI data as well as for in‐house multi‐echo acquisitions, confirming the higher performances of linear models. In particular, crRAKI with optimized parameters is shown to be superior to GRAPPA and rRAKI, with enhanced generalization capabilities. Notably, a new image quality metric, COBRAI, was proposed and shown to be able to rate the presence of structured residual artifacts observed in the reconstructions, enabling to better compare model performances. The presented results also highlight the significance of ACS size in all models, with crRAKI, which has fewer parameters, requiring less training data. The proposed methods could prove very useful for optimizing and comparing other parallel imaging reconstruction methods using machine learning approaches.

### 4.2 Nonlinearity

Our study showed that incorporating nonlinearities, such as in rRAKI, can be detrimental to model generalization, both using integrated and separated ACS modes. With a large number of parameters, there is a risk of network learning features from the ACS, such as contrast, which may not generalize well, especially in separated‐mode acquisitions where ACS do not share the same contrast as the undersampled k‐space. In multi‐echo sequences, the effects are more pronounced for larger echo times, which may be explained by increasing magnitude and phase discrepancy between the ACS and the undersampled k‐space. This contradicts the assumption that nonlinearities always improve reconstruction performance. The performance of rRAKI is closely related to the accuracy of the GRAPPA short‐connection in providing a reliable estimation. If the linear term shows poor performance, it is logical that rRAKI is also affected, as demonstrated in the ablation study. By increasing the degree of linearity with LeakyReLU [40] and the short‐connection loss, the rRAKI model becomes more efficient in modeling k‐space, but effectively produce artifacts, indicating that trained models do not generalize well to new data with nonlinearities. Similar observations have been reported in the crRAKI ablation study. These artifacts disappear with a linear model, leading to performances that are similar to those obtained using the single model loss term. These models exhibit implicit architectural regularization, which helps reduce the risk of overfitting and improves the robustness to contrast changes.

These findings suggest that activation functions such as ReLU should be used with care for rRAKI in the separated ACS acquisition mode. Even though the nonlinear rRAKI/crRAKI variant performance has better standard quantitative metrics than its linear variant in the integrated mode, COBRAI is higher, meaning that structural artifacts remain. The use of a short‐connection loss was not required in the training process, leading to consistent use of the MSE loss in k‐space for GRAPPA, rRAKI, and crRAKI linear models, avoiding the use of additional hyperparameters and reducing the computational requirements.

### 4.3 TVP and K‐Fold Cross‐Validation

In this study, a train‐validation partition scheme for training the models was applied. While this concept is standard in deep learning [41], it has also been applied in similar work recently to regularize the training stage by early stopping [15]. By partitioning the ACS, we simply applied early stopping to monitor the model's performance on the validation set and stopped training when its performance started to degrade. This approach helps prevent overfitting and encourages the model to converge towards optimal solutions. For a fair comparison, we consistently applied this strategy to regularize GRAPPA, rRAKI, and crRAKI.

As training and validating is done using a single ACS acquisition, fully sampled data are not needed to test the generalizability of the data. Our contribution is leveraging the use of K‐fold cross‐validation to assist in hyperparameter selection. Indeed, we demonstrate that the KSPACE‐MSE loss alone (using the ACS validation sets) can predict image quality metrics. This approach is a solution to the issue of heuristically determining the hyperparameters that were noted in an earlier study [30].

### 4.4 Kernel Sizes and Depths

We conducted optimization for kernel sizes and depths, specifically in a 2D case and for a limited set of size and depth combinations. Interestingly, our results confirm the validity of the sizes used in previous studies on GRAPPA and rRAKI [17, 21]. Furthermore, we demonstrate that several alternative hyperparameter sets can be selected. We utilized the same architectures as the ones employed in earlier studies to facilitate a comparison with the existing literature on GRAPPA and rRAKI.

The performance of neural networks depends on various factors, including the number of parameters and size of the ACS data. It is important to find the right balance between underfitting and overfitting. GRAPPA has shown competitive performance in some cases, but its limited flexibility in network design makes it less adaptable to changes in ACS size and number of receiver coils. Using a K‐fold cross‐validation strategy can help to determine the optimal network architecture size. As highlighted in previous studies [30, 42], additional self‐consistency is required along with calibration consistency in highly accelerated cases. We found that an optimal architecture can achieve satisfactory reconstruction in accelerated scenarios with smaller ACS sizes without requiring additional self‐consistency. Furthermore, we propose that iRAKI performance can be improved using crRAKI estimates as initializations.

The chosen crRAKI model was more compact than the GRAPPA model. The residual section is equivalent to the coil‐by‐coil SMASH [43] implementation, in parallel with a cRAKI section with three linear filters in a cascade. In our experiments, we did not observe any instability in learning crRAKI, such as divergence, which may be expected when learning cascade filters. To further reduce this risk, the first two layers can be regularized by imposing a unit norm to improve stability without any expected loss of performance.

### 4.5 ACS and Timing

We also evaluated the influence of ACS lines on the quality of the reconstructed images at varying acceleration rates. As expected, all methods in our study showed improved performance with increased ACS lines. Interestingly, the crRAKI(L) method demonstrated consistent results, even when fewer ACS lines were used, and regardless of the ACS acquisition mode, indicating a better stability to a low amount of training data.

On average, it takes between 15 and 30 s to train each task‐specific model in our current implementation, in which there is room for improvement in computation efficiency using parallelization. These timings align with those reported for RAKI and are significantly lower than those reported for iRAKI. Self‐consistency is more demanding in terms of computing resources than strategies based on using only the ACS, which is one advantage of the proposed approach compared to earlier works [30]. These limited training times render the technique attractive in the time‐efficient separated ACS mode and feasible in a routine workflow without dead times, as in ACS only, training can be performed immediately after the ACS is acquired at the beginning of scan, while the acquisition of the undersampled lines is ongoing. The evaluation can then be rapidly performed using the trained network.

### 4.6 COBRAI

We proposed the COBRAI image quality metric to address the limitations of existing metrics in measuring the presence of artifacts visible on the residual map. COBRAI, similar to SSIM, calculates the correlation coefficient through local computations on patches or blocks, followed by an average norm. Supervised upsampling in k‐space reconstruction aims to minimize the mean squared error (MSE) between the simulated data and target pairs obtained from the low‐resolution ACS data. This optimization objective simultaneously maximizes widely adopted evaluation metrics such as the peak signal‐to‐noise ratio (PSNR) and structural similarity index measure (SSIM). However, it is important to recognize that widely used metrics such as PSNR, SSIM, and NMAE have inherent limitations in capturing perceptually relevant features.

While COBRAI metrics are useful in evaluating performance, it is important to consider that extreme corruption may not always be fully captured. Therefore, a comprehensive analysis that combines multiple metrics, such as NRMSE, SSIM, and PSNR, is recommended for a more thorough evaluation. Despite its simplicity, COBRAI is effective in summarizing a specific type of reconstruction artifact and may be included in future DL‐MRI studies. Additionally, COBRAI can be incorporated into composite cost functions when training DL models, potentially reducing artifacts and opening new possibilities to improve reconstructions.

### 4.7 Improvements and Practical Implications

The model architecture was not thoroughly examined in the present study. Indeed, it was done for 2D to determine the hyperparameters, and performances in terms of image metrics were verified for a larger R, for various sequence types and coil setups. However, there might exist different hyperparameters more specific to these situations. This presents an opportunity to further optimize the performance of the model through a comprehensive exploration of its architecture by utilizing the presented hyperparameter optimization techniques. Although time‐consuming, this operation could be carried out for each patient, contrast and experimental set‐up. More optimally, it could also be achieved by including all these situations in a single hyper‐parameter selection procedure.

To the best of our knowledge, this is the first feasibility study of scan‐specific DL strategies with a focus on generalizability for both magnitude‐ and phase‐based applications in 2D. Using crRAKI, we have shown that 2D T2 W spin‐echo scans can be accelerated up to 4‐5 without noticeable artifacts, whereas $R_{y}$ = 2 is often used in practice, reducing the total scan time to 1 min. To address the issue of SNR loss expected when scan time is reduced, denoising approaches, including the use of image‐domain DL models [44, 45], could be employed to enhance the image quality.

Here, we show an application on a regular Cartesian GRAPPA‐like undersampling pattern. Extension to other structured undersampling patterns, such as CAIPIRINHA [46], as well as to 3D is straightforward.

## 5 Conclusions

In this work, we presented methods to optimize scan‐specific models for parallel imaging reconstruction. Grid‐search with K‐fold cross‐validation enables selecting the hyperparameters objectively. Partitioning the ACS into training and validation sets can help regularize the models using early stopping. We show that using nonlinearity is detrimental, particularly with a low amount of ACS, demonstrating that linear models perform better in scan specific MRI reconstruction, while it is often thought that the strength of deep‐learning approaches comes from nonlinearity. After optimization, higher acceleration rates than the ones commonly used can be obtained. The crRAKI model has been found to outperform GRAPPA and rRAKI, especially in the separated ACS mode, as evidenced by the improved quantitative metrics and image quality in 2D for various sequence types, including some focusing on phase. The question whether the most efficient model, that is, a three‐layer linear complex convolution with a residual connection (linear crRAKI) is considered as a deep neural network arises. The quantification of structured residual artifacts with COBRAI suggests a complementary characterization to state‐of‐the‐art metrics, leading to more optimal k‐space undersampling, ACS acquisition and reconstruction models in scan‐specific strategies. Integrating residual‐artifact‐sensitive metrics such as COBRAI within objective cost functions as well as focusing on optimizing ACS data acquisition for training, especially in separated‐mode, may further reduce artifacts, provide a larger acceleration rate and offer new perspectives for transferring deep‐learning methodologies to parallel MRI.

## Author Contributions

Funding acquisition: T.A., L.D.R., and M.G.; data acquisition: S.N., L.D.R., and M.G.; study conceptualization, experiment design, and data analysis: S.N., T.A., and L.D.R.; drafting of the manuscript: S.N., T.A., and L.D.R.; review of the manuscript: all.

## Conflicts of Interest

S.N., T.A., and L.D.R. are listed as inventors in a patent filed related to the use of COBRAI metric (FR3153449/WO2025061399). The authors declare no other potential conflict of interests.

## Supporting information

NMR_bioMed_2024_Swetali_review_clean_version_supplementary.pdf

## Data Availability

The code will be made available upon publication (https://github.com/L2roche/cobrai_paper). The fastMRI dataset is publicly available such that the associated results presented here can be reproduced.
