# Supplementary material for: Insights on Scan‐Specific Deep‐Learning Strategies for Brain MRI Parallel Imaging Reconstruction
Source: NMR Biomed. 2025 Jun 22;38(8):e70079. doi: 10.1002/nbm.70079 (PMC12183417; doi:10.1002/nbm.70079)
Supplement: Supplementary file 1 — NMR_bioMed_2024_Swetali_review_clean_version_supplementary.pdf [file NBM-38-e70079-s001.pdf]

TABLE S1 List of valid models obtained through a grid search. To identify the most suitable set of hyperparameters, a grid search based on the K-fold cross-validation was performed and based on the mean squared error (MSE) metric in k-space the potential list of architecture was obtained. To determine whether the performance differences observed across different hyperparameters were statistically significant, we conducted a one-tailed Student's t-test, which enabled us to compare the average performance metric among various hyperparameters. The architecture was optimized for GRAPPA in 2D, using kernel sizes of $[n_{x,g}, n_{y,g}]$ = [3,2], [5,2], [5,4], [5,5], [7,2], [7,4], [9,2], and [9,4]. In 2D, to determine the hyperparameters for both rRAKI and crRAKI, all possible combinations were tested for the following: first layer size $[n_{x,1}, n_{y,1}]$ = [5,2] and [5,4], second layer size set to $[n_{x,2}, n_{y,2}]$ = [1,1], layer depths $2 \times n_{d,1}$ and $2 \times n_{d,2}$ = 32, 16 and 8, third layer size $[n_{x,3}, n_{y,3}]$ = [3,2] and [3,4], and residual layer size $[n_{x,g}, n_{y,g}]$ = [5,2] and [1,1]. This led to a total of 72 different architectures for both rRAKI and crRAKI. Only non-significantly different cases are reported. In the column labelled 'R', bold indicates the best solutions for a given R. As can seen, several kernel sizes can be selected for GRAPPA, the best being [5, 4] which is suitable for all acceleration rates and is a commonly-used kernel size. For rRAKI, several hyperparameters are possible, and the ones used (in bold) correspond to a situation suitable for several acceleration rates. For crRAKI, as can be seen, several kernel sizes and depths can be chosen, and we selected one with a smaller kernel size in the residual layer which is suitable for several acceleration rates.

Model	KERNEL SIZE	KERNEL DEPTH	R
**GRAPPA**	[3, 4]	-	**2**, **3**, 4, **6**
	**[5, 4]**	-	2, 3, **4**, **5**, 6
	[7, 4]	-	6
**rRAKI**	[5 2], [1 1], [3 2], [1 1]	32, 32	2, 3, 4, 5
	[5 2], [1 1], [3 2], [5 2]	8, 8	2, **3**, 4, 6
	[5 2], [1 1], [3 2], [5 2]	8, 16	**2**, 3, 4, 5, 6
	[5 2], [1 1], [3 2], [5 2]	8, 32	2, 3, 4, 5
	[5 2], [1 1], [3 2], [5 2]	16, 8	2, 3, 4, 6
	[5 2], [1 1], [3 2], [5 2]	16, 16	2, 3, 4, 5, 6
	[5 2], [1 1], [3 2], [5 2]	16, 32	2, 3, 4, 5
	**[5 2], [1 1], [3 2], [5 2]**	**32, 8**	2, 3, 4, **6**
	[5 2], [1 1], [3 2], [5 2]	32, 16	2, 3, 4, 5, 6
	[5 2], [1 1], [3 2], [5 2]	32, 32	2, 3, **4**, **5**
**crRAKI**	**[5 2], [1 1], [3 2], [1 1]**	**16, 32**	2, 3, 4, 5
	[5 2], [1 1], [3 2], [1 1]	32, 32	2, 3, 4, 5
	[5 2], [1 1], [3 2], [5 2]	8, 8	2, 3, 4, 5, 6
	[5 2], [1 1], [3 2], [5 2]	8, 16	2, 3, 4, 5
	[5 2], [1 1], [3 2], [5 2]	8, 32	**2**, **3**, **4**, **5**
	[5 2], [1 1], [3 2], [5 2]	16, 8	2, 3, 4, 5, 6
	[5 2], [1 1], [3 2], [5 2]	16, 16	2, 3, 4, 5
	[5 2], [1 1], [3 2], [5 2]	16, 32	2, 3, 4, 5
	[5 2], [1 1], [3 2], [5 2]	32, 8	2, 3, 4, 5, **6**
	[5 2], [1 1], [3 2], [5 2]	32, 16	2, 3, 4, 5, 6
	[5 2], [1 1], [3 2], [5 2]	32, 32	2, 3, 4, 5

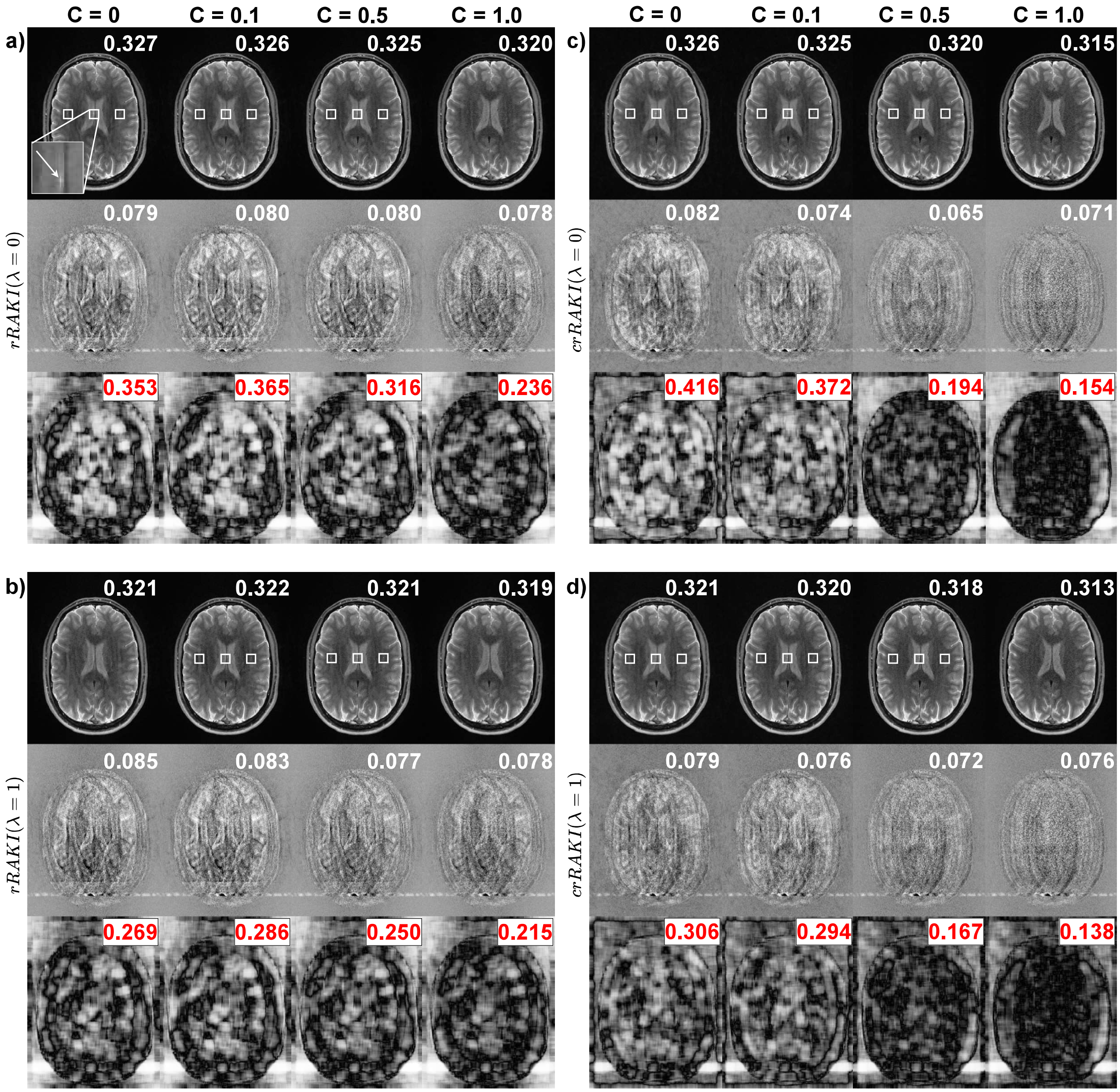

FIGURE S1 Illustration of the effects of non-linearities for R_y=5 on the T2W Spin-Echo data with N_y^{acs}=40 (R_{eff}=4.43) using rRAKI (a, b) and crRAKI (c, d) with LeakyReLU activation function by varying the coefficient parameter c, where the top set corresponds to $\lambda = 0$ (a, c) and the bottom set corresponds to adding a short-connection loss with $\lambda = 1$ (b, d). For each set, the first row if the reconstructed magnitude image, the second row is the residual, the third row is the COBRA map. The numbers on the top-right corners are respectively the Blur metrics, the mean RMSE, the COBRAI taken over a brain mask. As can be seen, both structured artifacts decrease with increasing c, associated with a reduction of RMSE and COBRAI. The addition of a short-connection loss enhances the results, enforcing linearity of this section of the models. Artifactual dots are noticeable visually (boxes) for up to $c < 1$ both for $\lambda = 0$ and $\lambda = 1$. There is a decrease in the intensity of these artifacts when $\lambda = 1$, but they persist in the final reconstruction. Overall, best results are obtained when fully-linear models are used, regardless the addition of a short-connection loss.

TABLE S2 Effect of non-linearities using rRAKI on T2W Spin-Echo data with N_y^{acs}=40 and R_y=5 with LeakyReLU activation function by varying the parameter c between 0 and 1, with λ = 0 or 1. K-FOLD cross-validation was performed to determine the optimal parameters based on K-SPACE Mean Squared Error (MSE), which corresponds to the model loss on the validation set (mean over all folds). The model loss decreases with increasing c values for both λ conditions. For a given c, the MSE loss was lower when λ = 1 compared to λ = 0 when c < 1, indicating that rRAKI performed better with the additional short-connection loss term. However, the best performance was observed when c = 1 (linear model) and λ = 0. The final reconstruction performance of the trained model was evaluated using Normalized Root Mean Squared Error (NRMSE), Normalized Mean Absolute Error (NMAE) and PSNR, yielding the best results for λ = 0 and c = 1. Metrics are better for c = 1, signifying linear models are the best. We conclude that non-linearity degrades the performances and that an additional loss term is then not needed. The best-performing results are denoted in **bold**, and metrics without significatively different mean are indicated in *italics*.

λ	C	KSPACE-MSE (1e-7)	NRMSE	NMAE	SSIM	PSNR	Blur Metrics	COBRAI
**REF**		**K-FOLD**					0.340 ± 0.065	
	0.000	4.818 ± 5.103	0.125 ± 0.049	0.114 ± 0.042	0.943 ± 0.026	34.368 ± 2.309	0.338 ± 0.064	0.365 ± 0.092
	0.010	4.750 ± 5.037	0.124 ± 0.049	0.113 ± 0.043	0.943 ± 0.026	34.398 ± 2.364	0.338 ± 0.065	0.364 ± 0.094
	0.100	4.342 ± 4.657	0.121 ± 0.048	0.110 ± 0.042	0.945 ± 0.026	34.719 ± 2.334	0.338 ± 0.064	0.354 ± 0.091
	0.200	3.710 ± 3.911	0.115 ± 0.046	0.106 ± 0.040	0.946 ± 0.026	35.158 ± 2.297	0.337 ± 0.065	0.340 ± 0.086
0	0.300	3.165 ± 3.300	0.111 ± 0.044	0.102 ± 0.039	0.948 ± 0.025	35.501 ± 2.232	0.337 ± 0.065	0.327 ± 0.081
	0.500	2.272 ± 2.302	0.106 ± 0.041	0.098 ± 0.037	**0.949 ± 0.025**	35.905 ± 2.192	0.336 ± 0.065	0.306 ± 0.072
	0.700	1.476 ± 1.325	0.104 ± 0.039	0.097 ± 0.037	0.948 ± 0.026	36.069 ± 2.148	0.335 ± 0.065	0.291 ± 0.068
	0.900	0.767 ± 0.486	0.100 ± 0.039	0.095 ± 0.038	0.947 ± 0.028	36.419 ± 2.223	0.333 ± 0.066	0.264 ± 0.056
	1.000	**0.602 ± 0.357**	**0.099 ± 0.040**	**0.094 ± 0.039**	0.947 ± 0.029	**36.556 ± 2.254**	0.333 ± 0.066	0.250 ± 0.050
	0.000	1.718 ± 1.338	0.113 ± 0.043	0.107 ± 0.042	0.940 ± 0.030	35.426 ± 2.215	0.333 ± 0.065	0.294 ± 0.071
	0.010	1.679 ± 1.240	0.112 ± 0.043	0.106 ± 0.042	0.940 ± 0.030	35.452 ± 2.238	0.333 ± 0.065	0.294 ± 0.071
	0.100	1.663 ± 1.328	0.110 ± 0.042	0.104 ± 0.042	0.942 ± 0.029	35.647 ± 2.224	0.333 ± 0.065	0.288 ± 0.069
	0.200	1.596 ± 1.228	0.108 ± 0.043	0.102 ± 0.042	0.943 ± 0.030	35.829 ± 2.237	0.333 ± 0.065	0.282 ± 0.067
1	0.300	1.487 ± 1.159	0.107 ± 0.043	0.101 ± 0.041	0.944 ± 0.029	35.952 ± 2.181	0.333 ± 0.066	0.278 ± 0.064
	0.500	1.275 ± 0.923	0.103 ± 0.041	0.097 ± 0.04	0.946 ± 0.029	36.229 ± 2.225	0.333 ± 0.066	0.271 ± 0.062
	0.700	1.027 ± 0.710	0.101 ± 0.041	0.096 ± 0.04	0.946 ± 0.029	36.439 ± 2.214	0.332 ± 0.066	0.263 ± 0.058
	0.900	0.703 ± 0.419	0.100 ± 0.041	0.095 ± 0.041	0.945 ± 0.030	*36.537 ± 2.244*	0.331 ± 0.066	0.251 ± 0.053
	1.000	*0.622 ± 0.363*	0.101 ± 0.043	0.097 ± 0.043	0.944 ± 0.032	36.451 ± 2.314	**0.331** ± *0.066*	**0.248 ± 0.051**

Standard deviations are reported over all slices.

TABLE S3 Effect of non-linearities using crRAKI on T2W Spin-Echo data with N_y^{acs}=40 and R_y=5 with LeakyReLU activation function by varying the parameter c between 0 and 1, with λ = 0 or 1. K-FOLD cross-validation was performed to determine the optimal parameters based on K-SPACE Mean Squared Error (MSE), which corresponds to the model loss on the validation set (mean over all folds). The model loss decreases with increasing c values for both λ conditions. For a given c, the MSE loss was lower when $\lambda = 1$ compared to $\lambda = 0$ when $c < 1$, indicating that crRAKI performed better with the additional short-connection loss term. However, the best performance was observed when c = 1 (linear model) and λ = 0. The final reconstruction performance of the trained model was evaluated using Normalized Root Mean Squared Error (NRMSE), Normalized Mean Absolute Error (NMAE) and PSNR, yielding the best results for λ = 0 and c = 1. Metrics are better for c = 1, signifying linear models are the best. We conclude that non-linearity degrades the performances and that an additional loss term is then not needed. The best-performing results are denoted in **bold**, and metrics without significatively different mean are indicated in *italics*.

λ	C	KSPACE-MSE (1e-7)	NRMSE	NMAE	SSIM	PSNR	Blur Metrics	COBRAI
**REF**		**K-FOLD**					0.340 ± 0.065	
	0.000	7.003 ± 2.630	0.120 ± 0.045	0.112 ± 0.040	0.948 ± 0.022	34.572 ± 2.229	0.339 ± 0.068	0.400 ± 0.091
	0.010	7.155 ± 2.865	0.105 ± 0.037	0.088 ± 0.034	0.972 ± 0.026	35.815 ± 2.027	0.311 ± 0.068	0.370 ± 0.109
	0.100	5.872 ± 2.217	0.096 ± 0.035	0.082 ± 0.033	0.974 ± 0.026	36.568 ± 2.144	0.310 ± 0.068	0.349 ± 0.106
	0.200	4.840 ± 1.768	0.089 ± 0.033	0.076 ± 0.033	0.975 ± 0.027	37.392 ± 2.130	0.310 ± 0.067	0.325 ± 0.104
0	0.300	4.239 ± 1.558	0.083 ± 0.032	0.071 ± 0.033	0.975 ± 0.028	38.009 ± 2.298	0.310 ± 0.067	0.305 ± 0.104
	0.500	2.735 ± 0.970	0.075 ± 0.031	0.065 ± 0.033	**0.976 ± 0.029**	38.982 ± 2.421	0.309 ± 0.067	0.263 ± 0.100
	0.700	1.876 ± 0.633	**0.071 ± 0.030**	**0.063 ± 0.033**	0.975 ± 0.029	39.555 ± 2.556	0.308 ± 0.067	0.221 ± 0.098
	0.900	1.210 ± 0.415	0.071 ± 0.031	0.063 ± 0.033	0.973 ± 0.030	**39.558 ± 2.649**	0.307 ± 0.066	0.193 ± 0.098
	1.000	**1.105 ± 0.410**	0.072 ± 0.031	0.064 ± 0.033	0.972 ± 0.030	39.437 ± 2.672	**0.307 ± 0.067**	**0.188 ± 0.098**
	0.000	5.256 ± 2.027	0.114 ± 0.043	0.112 ± 0.042	0.938 ± 0.026	35.148 ± 2.071	0.330 ± 0.062	0.341 ± 0.083
	0.010	4.954 ± 1.842	0.092 ± 0.030	0.079 ± 0.025	0.972 ± 0.016	36.971 ± 1.922	0.308 ± 0.066	0.295 ± 0.078
	0.100	4.190 ± 1.586	0.087 ± 0.028	0.075 ± 0.025	0.973 ± 0.017	37.485 ± 1.885	0.307 ± 0.066	0.279 ± 0.075
	0.200	3.468 ± 1.193	0.101 ± 0.038	0.099 ± 0.038	0.945 ± 0.025	36.306 ± 2.001	0.330 ± 0.063	0.299 ± 0.069
1	0.300	2.881 ± 0.951	0.096 ± 0.036	0.095 ± 0.036	0.947 ± 0.025	36.725 ± 1.982	0.330 ± 0.063	0.280 ± 0.064
	0.500	2.123 ± 0.653	0.089 ± 0.034	0.088 ± 0.034	0.950 ± 0.025	37.387 ± 2.007	0.329 ± 0.065	0.245 ± 0.052
	0.700	1.578 ± 0.466	0.087 ± 0.033	0.085 ± 0.034	0.950 ± 0.026	37.707 ± 2.105	0.329 ± 0.065	0.214 ± 0.043
	0.900	1.318 ± 0.397	0.088 ± 0.034	0.086 ± 0.035	0.947 ± 0.027	37.643 ± 2.194	0.327 ± 0.066	0.193 ± 0.037
	1.000	1.280 ± 0.395	0.088 ± 0.035	0.087 ± 0.035	0.946 ± 0.028	37.569 ± 2.233	0.327 ± 0.066	0.190 ± 0.036

Standard deviations are reported over all slices.

TABLE S4 Mean and standard deviations (over 300 slices) for NRMSE, NMAE, SSIM, PSNR, Blur metrics and COBRAI for the tested reconstruction methods for FLAIR, T1W, T1POST and T2W from the fastMRI dataset at R = 4 for N_y^{acs}=24. The method with the best metric **in bold** is compared with the other methods. Metrics without statistical significance (p > 0.05 - 5% level) are indicated in *italics*. When less ACS (24) are used for training, rRAKI performs well only for T1W images, while the performance comparison with GRAPPA and crRAKI yielded mixed results. crRAKI always outperformed GRAPPA and rRAKI in terms of COBRAI, indicating less reconstruction artifacts.

TYPE	METHOD	NRMSE	NMAE	SSIM	PSNR	BLUR METRICS	COBRAI
FLAIR	**Reference**					0.309 ± 0.046	
	GRAPPA(TR)	0.100 ± 0.037	0.092 ± 0.035	0.893 ± 0.062	33.171 ± 3.298	0.336 ± 0.034	0.179 ± 0.062
	GRAPPA(TVP)	0.102 ± 0.026	0.070 ± 0.026	0.886 ± 0.062	32.423 ± 3.309	**0.331 ± 0.033**	0.181 ± 0.066
	crRAKI(L)	0.099 ± 0.038	0.090 ± 0.036	**0.895 ± 0.059**	**33.221 ± 3.353**	0.333 ± 0.033	**0.160 ± 0.066**
	crRAKI(NL)	0.106 ± 0.03	0.096 ± 0.028	0.882 ± 0.061	32.372 ± 3.018	0.339 ± 0.032	0.208 ± 0.079
	rRAKI(L)	0.103 ± 0.035	0.093 ± 0.034	0.892 ± 0.057	32.600 ± 3.137	0.340 ± 0.030	0.208 ± 0.076
	rRAKI(NL)	**0.097 ± 0.027**	**0.067 ± 0.026**	*0.895 ± 0.058*	32.557 ± 3.07	0.351 ± 0.032	0.237 ± 0.085
T1W	**Reference**					0.397 ± 0.066	
	GRAPPA(TR)	0.110 ± 0.043	0.107 ± 0.039	0.870 ± 0.077	34.770 ± 3.354	*0.393 ± 0.078*	0.196 ± 0.066
	GRAPPA(TVP)	0.118 ± 0.042	0.094 ± 0.04	0.872 ± 0.077	34.016 ± 3.359	0.401 ± 0.077	0.217 ± 0.073
	crRAKI(L)	0.106 ± 0.044	0.102 ± 0.041	0.877 ± 0.075	35.137 ± 3.501	**0.393 ± 0.079**	**0.185 ± 0.076**
	crRAKI(NL)	**0.089 ± 0.033**	0.088 ± 0.031	**0.908 ± 0.054**	**36.249 ± 2.430**	0.419 ± 0.070	0.211 ± 0.075
	rRAKI(L)	0.104 ± 0.044	0.101 ± 0.04	0.882 ± 0.072	35.221 ± 3.418	0.405 ± 0.08	0.219 ± 0.078
	rRAKI(NL)	0.093 ± 0.034	**0.075 ± 0.032**	0.901 ± 0.061	35.888 ± 2.885	0.421 ± 0.076	0.229 ± 0.076
T1POST	**Reference**					0.412 ± 0.068	
	GRAPPA(TR)	**0.060 ± 0.031**	0.057 ± 0.029	**0.936 ± 0.062**	**38.612 ± 3.483**	0.453 ± 0.094	0.173 ± 0.062
	GRAPPA(TVP)	0.082 ± 0.033	0.061 ± 0.032	0.922 ± 0.065	36.107 ± 3.581	0.445 ± 0.090	0.165 ± 0.062
	crRAKI(L)	0.062 ± 0.032	0.057 ± 0.030	0.933 ± 0.063	38.509 ± 3.580	**0.444 ± 0.093**	**0.126 ± 0.058**
	crRAKI(NL)	0.082 ± 0.026	0.076 ± 0.023	0.909 ± 0.062	35.814 ± 2.641	0.430 ± 0.075	0.164 ± 0.074
	rRAKI(L)	0.066 ± 0.031	0.061 ± 0.029	0.933 ± 0.061	37.785 ± 3.398	0.450 ± 0.088	0.165 ± 0.069
	rRAKI(NL)	0.072 ± 0.032	**0.055 ± 0.032**	0.928 ± 0.061	37.042 ± 3.102	0.450 ± 0.086	0.169 ± 0.074
T2W	**Reference**					0.365 ± 0.053	
	GRAPPA(TR)	**0.062 ± 0.030**	0.062 ± 0.028	0.956 ± 0.044	**39.531 ± 3.226**	0.393 ± 0.043	0.145 ± 0.049
	GRAPPA(TVP)	0.072 ± 0.026	**0.058 ± 0.027**	0.952 ± 0.045	38.058 ± 3.027	**0.387 ± 0.042**	0.140 ± 0.053
	crRAKI(L)	0.064 ± 0.030	0.062 ± 0.029	**0.956 ± 0.045**	39.390 ± 3.332	0.389 ± 0.043	**0.110 ± 0.049**
	crRAKI(NL)	0.092 ± 0.025	0.090 ± 0.024	0.930 ± 0.045	35.925 ± 2.567	0.38 ± 0.038	0.155 ± 0.066
	rRAKI(L)	0.072 ± 0.03	0.069 ± 0.028	0.951 ± 0.045	38.188 ± 3.286	0.392 ± 0.043	0.142 ± 0.055
	rRAKI(NL)	0.077 ± 0.029	0.061 ± 0.030	0.950 ± 0.045	37.518 ± 3.273	0.394 ± 0.042	0.154 ± 0.060

Abbreviations: GRAPPA(TR): GRAPPA with Tikhonov Regularization, GRAPPA (TVP): GRAPPA with Train Validation Partition, L: Linear ($\lambda = 0, c = 1$), NL: Non-linear ($\lambda = 1, c = 0$)

TABLE S5 Mean and standard deviations (over 300 slices) for NRMSE, NMAE, SSIM, PSNR, Blur metrics and COBRAI for the tested reconstruction methods for FLAIR, T1W, T1POST and T2W from the fastMRI dataset at R = 4 for N_y^{acs}=40. The method with the best metric **in bold** is compared with the other methods. Metrics without statistical significance (p > 0.05 - 5% level) are indicated in *italics*. While rRAKI has better image quality metrics (NRMSE, NMAE, SSIM, PSNR) in the case of T1W and FLAIR images, this is not the case for T1POST and T2W.

TYPE	METHOD	NRMSE	NMAE	SSIM	PSNR	BLUR METRICS	COBRAI
FLAIR	GRAPPA(TR)	0.093 ± 0.035	0.086 ± 0.033	0.903 ± 0.054	33.723 ± 3.312	0.338 ± 0.033	0.171 ± 0.063
	GRAPPA(TVP)	0.089 ± 0.026	0.062 ± 0.024	0.900 ± 0.055	33.489 ± 3.298	**0.335 ± 0.033**	0.166 ± 0.064
	crRAKI(L)	0.093 ± 0.036	0.085 ± 0.034	0.904 ± 0.054	33.756 ± 3.339	0.337 ± 0.033	**0.162 ± 0.065**
	crRAKI(NL)	0.088 ± 0.026	0.080 ± 0.024	0.907 ± 0.052	33.986 ± 2.784	0.349 ± 0.033	0.179 ± 0.059
	rRAKI(L)	0.095 ± 0.036	0.086 ± 0.034	0.899 ± 0.054	33.392 ± 3.164	0.339 ± 0.03	0.185 ± 0.072
	rRAKI(NL)	**0.082 ± 0.025**	**0.057 ± 0.023**	**0.912 ± 0.050**	**34.020 ± 2.981**	0.356 ± 0.034	0.209 ± 0.072
T1W	GRAPPA(TR)	0.098 ± 0.041	0.096 ± 0.038	0.884 ± 0.071	35.714 ± 3.449	0.399 ± 0.079	0.189 ± 0.067
	GRAPPA(TVP)	0.097 ± 0.037	0.079 ± 0.034	0.884 ± 0.072	35.573 ± 3.419	**0.399 ± 0.079**	0.190 ± 0.068
	crRAKI(L)	0.098 ± 0.041	0.095 ± 0.038	0.885 ± 0.073	35.779 ± 3.540	0.400 ± 0.080	**0.183 ± 0.071**
	crRAKI(NL)	0.077 ± 0.034	0.076 ± 0.032	0.926 ± 0.051	37.722 ± 2.817	0.426 ± 0.074	0.205 ± 0.080
	rRAKI(L)	0.088 ± 0.038	0.087 ± 0.036	0.902 ± 0.062	36.570 ± 3.228	0.413 ± 0.077	0.198 ± 0.071
	rRAKI(NL)	**0.075 ± 0.03**	**0.062 ± 0.027**	**0.923 ± 0.051**	**37.563 ± 2.898**	0.430 ± 0.078	0.216 ± 0.074
T1POST	GRAPPA(TR)	**0.056 ± 0.031**	0.053 ± 0.028	0.939 ± 0.058	39.185 ± 3.505	0.453 ± 0.094	0.155 ± 0.058
	GRAPPA(TVP)	0.061 ± 0.030	0.048 ± 0.030	0.934 ± 0.060	38.402 ± 3.428	**0.445 ± 0.090**	0.140 ± 0.059
	crRAKI(L)	0.058 ± 0.031	0.054 ± 0.029	0.938 ± 0.06	**39.020 ± 3.639**	0.449 ± 0.093	**0.131 ± 0.058**
	crRAKI(NL)	0.065 ± 0.027	0.061 ± 0.025	0.93 ± 0.058	37.82 ± 2.851	0.442 ± 0.082	0.137 ± 0.064
	rRAKI(L)	0.060 ± 0.031	0.056 ± 0.029	0.938 ± 0.058	38.641 ± 3.405	0.448 ± 0.089	0.146 ± 0.063
	rRAKI(NL)	0.059 ± 0.03	**0.046 ± 0.029**	**0.940 ± 0.056**	38.648 ± 3.079	0.454 ± 0.089	0.151 ± 0.064
T2W	GRAPPA(TR)	0.060 ± 0.029	0.060 ± 0.028	0.958 ± 0.042	39.879 ± 3.250	0.394 ± 0.043	0.137 ± 0.047
	GRAPPA(TVP)	0.063 ± 0.025	**0.051 ± 0.025**	0.956 ± 0.042	39.180 ± 3.078	**0.389 ± 0.042**	0.122 ± 0.050
	crRAKI(L)	**0.060 ± 0.029**	0.059 ± 0.028	**0.959 ± 0.043**	**39.897 ± 3.385**	0.392 ± 0.043	**0.117 ± 0.048**
	crRAKI(NL)	0.073 ± 0.025	0.071 ± 0.024	0.947 ± 0.043	38.011 ± 2.567	0.388 ± 0.04	0.123 ± 0.052
	rRAKI(L)	0.065 ± 0.029	0.064 ± 0.027	0.955 ± 0.043	39.107 ± 3.22	0.392 ± 0.042	0.125 ± 0.049
	rRAKI(NL)	0.063 ± 0.025	0.052 ± 0.026	0.957 ± 0.042	39.089 ± 2.988	0.396 ± 0.042	0.132 ± 0.050

Abbreviations: GRAPPA(TR): GRAPPA with Tikhonov Regularization, GRAPPA (TVP): GRAPPA with Train Validation Partition, L: Linear ($\lambda = 0, c = 1$), NL: Non-linear ($\lambda = 1, c = 0$)

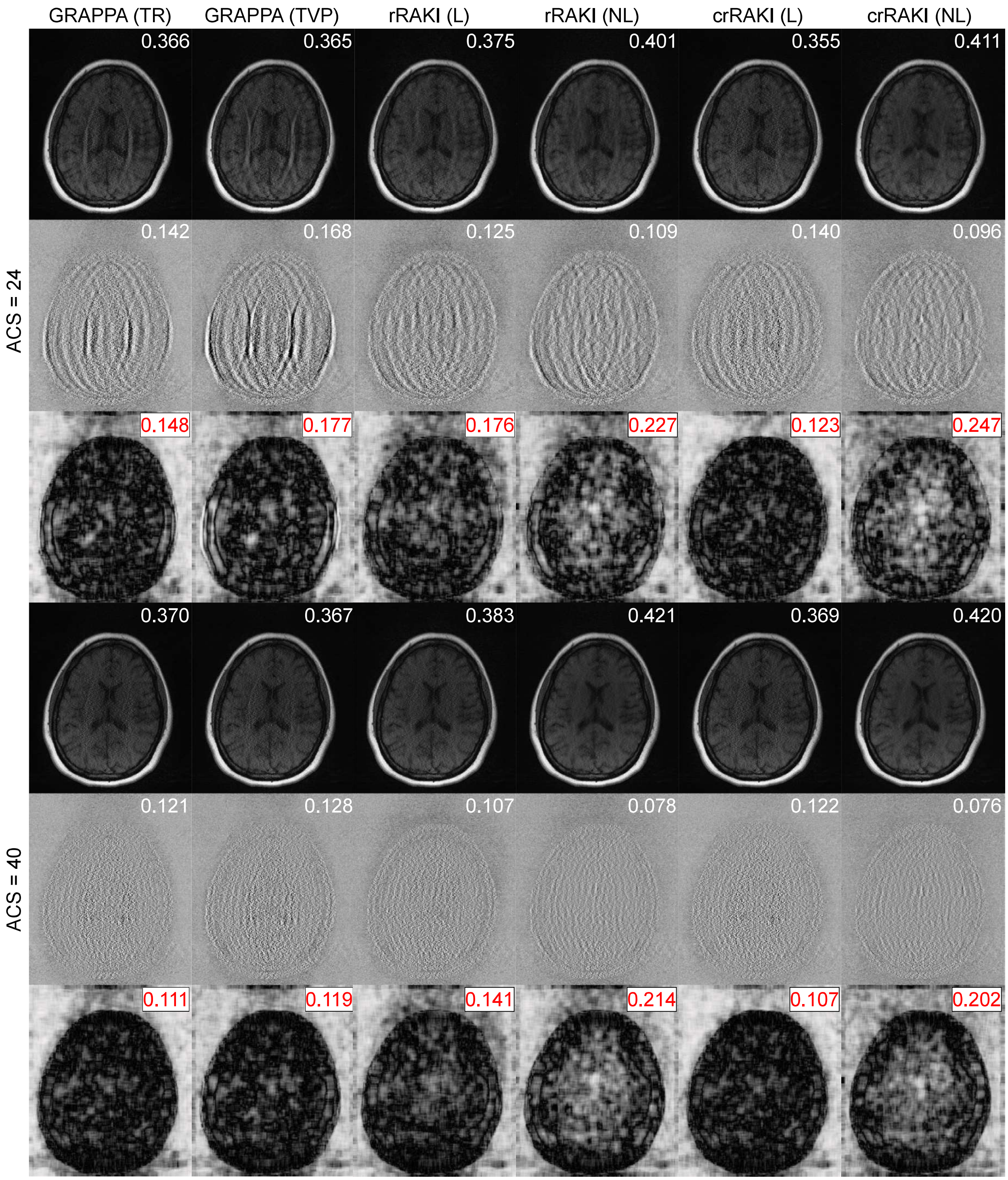

FIGURE S2 Comparison of the reconstruction for FastMRI T1 dataset id 200_6000353 slice index 0 for R_y=4 and two ACS size (24 and 40, R_{eff}=3.16 and 2.78, respectively). For each ACS set, the first row if the reconstructed magnitude image, the second row is the residual, the third row is the COBRA map. The numbers on the top-right corners are respectively the Blur metrics, the mean RMSE, the COBRAI taken over a brain mask. The crRAKI NL is the model that provides the smaller RMSE for both ACS, but with a larger amount of structured residual artifacts than its Linear counterpart.

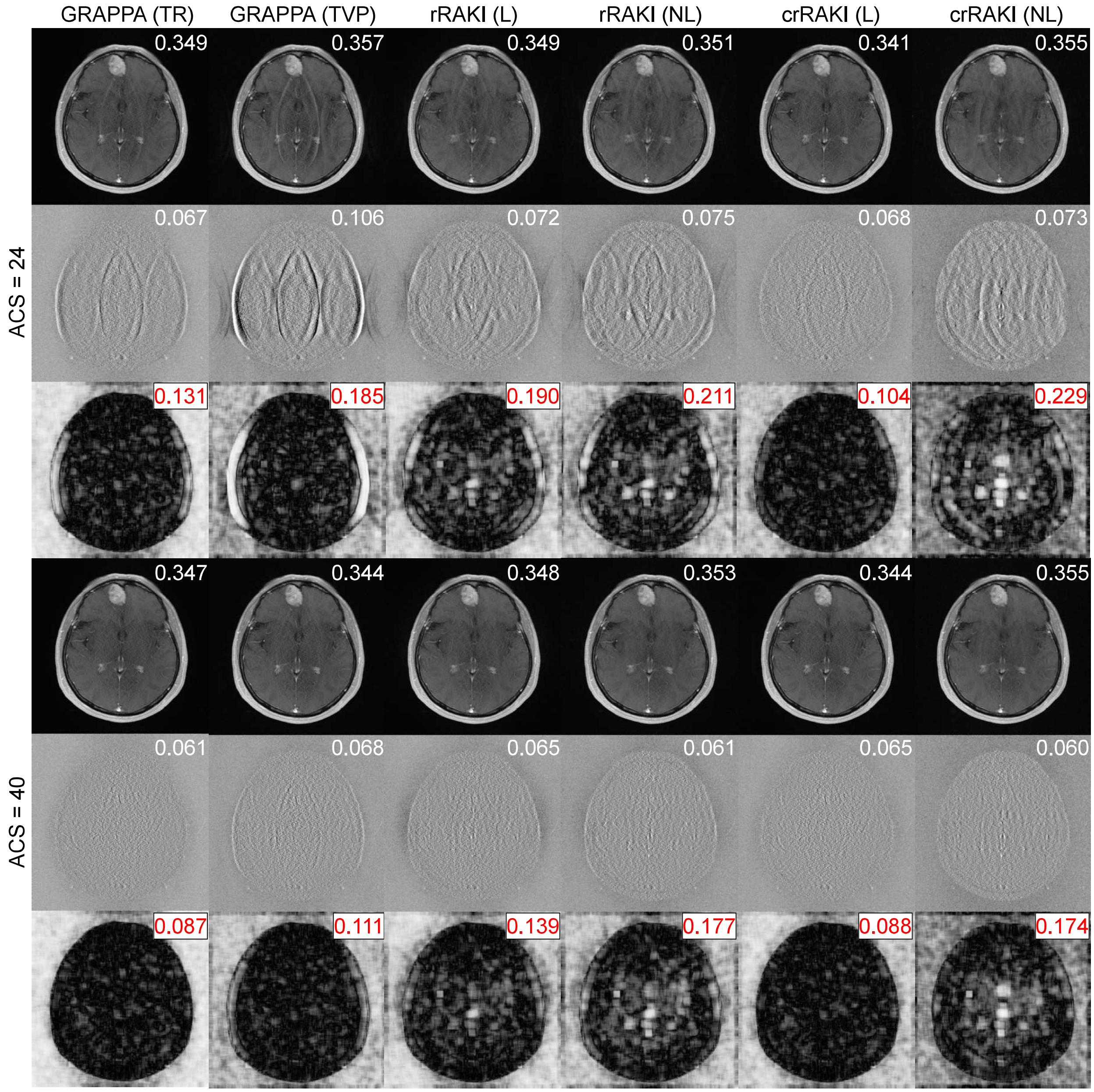

FIGURE S3 Comparison of the reconstruction for FastMRI T1POST dataset id 200_6002065 slice index 0 for R_y=4 and two ACS sizes (24 and 40, R_{eff}=3.27 and 2.91, respectively). For each ACS set, the first row if the reconstructed magnitude image, the second row is the residual, the third row is the COBRA map. The numbers on the top-right corners are respectively the Blur metrics, the mean RMSE, the COBRAI taken over a brain mask. GRAPPA with Tikhonov Regularization (TR) and Train-Validation Partition (TVP), as well as rRAKI Linear (L) and Non-Linear (NL) perform well only when ACS size is large enough. rRAKI (NL) for N_y^{acs}=40 appears to be the one with lower NRMSE, but with significant structured residual artifacts. The crRAKI Linear method demonstrates superior capability in reconstructing high-quality images with enhanced fidelity and accuracy.

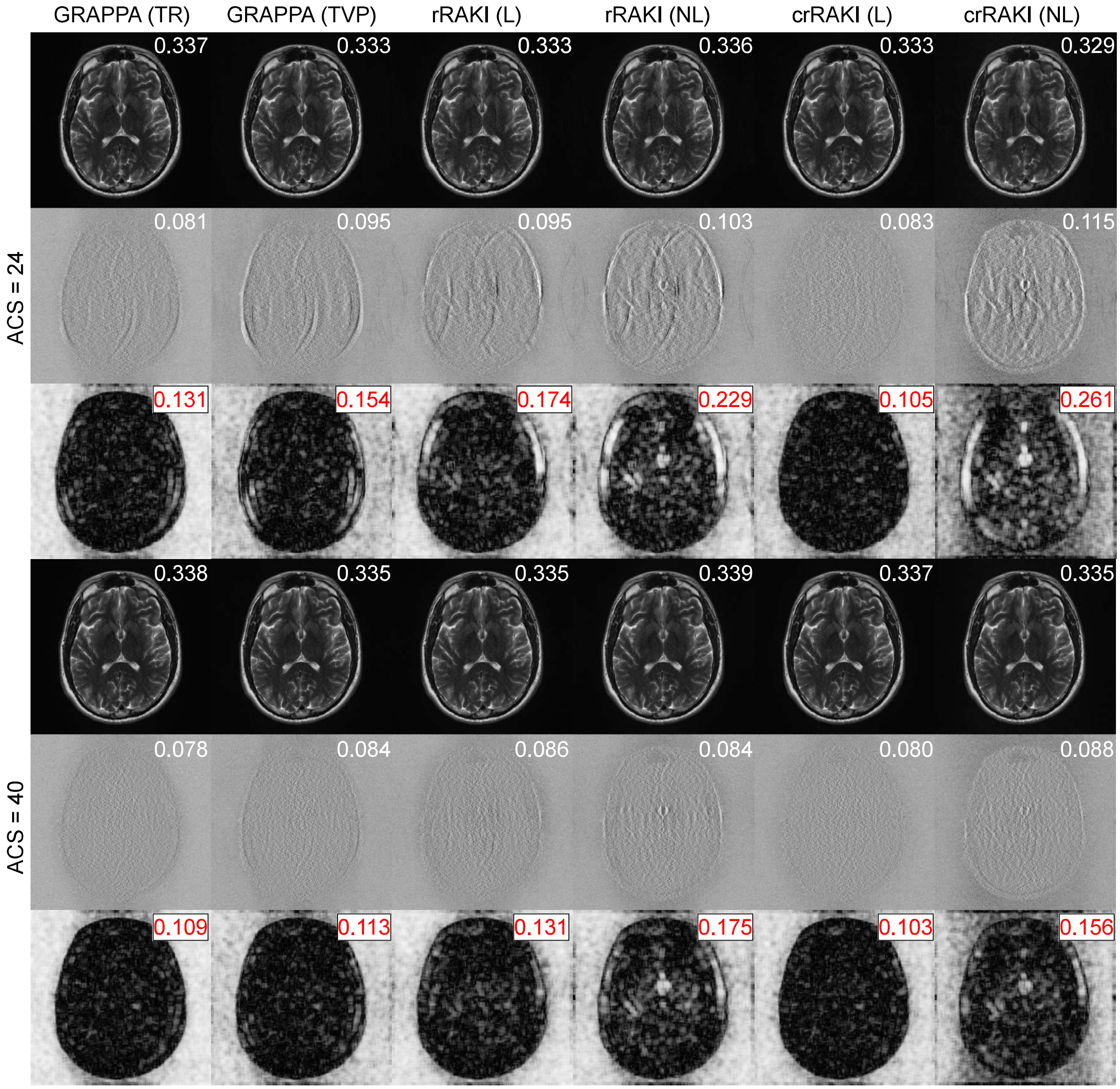

FIGURE S4 Comparison of the reconstruction for FastMRI T2 dataset id 200_6000275 slice index 0 for R_y=4 and two ACS sizes (24 and 40, R_{eff}=3.38 and 3.07, respectively). For each ACS set, the first row if the reconstructed magnitude image, the second row is the residual, the third row is the COBRA map. The numbers on the top-right corners are respectively the Blur metrics, the mean RMSE, the COBRAI taken over a brain mask. The crRAKI Linear method demonstrates superior capability in reconstructing high-quality images with enhanced fidelity and accuracy.

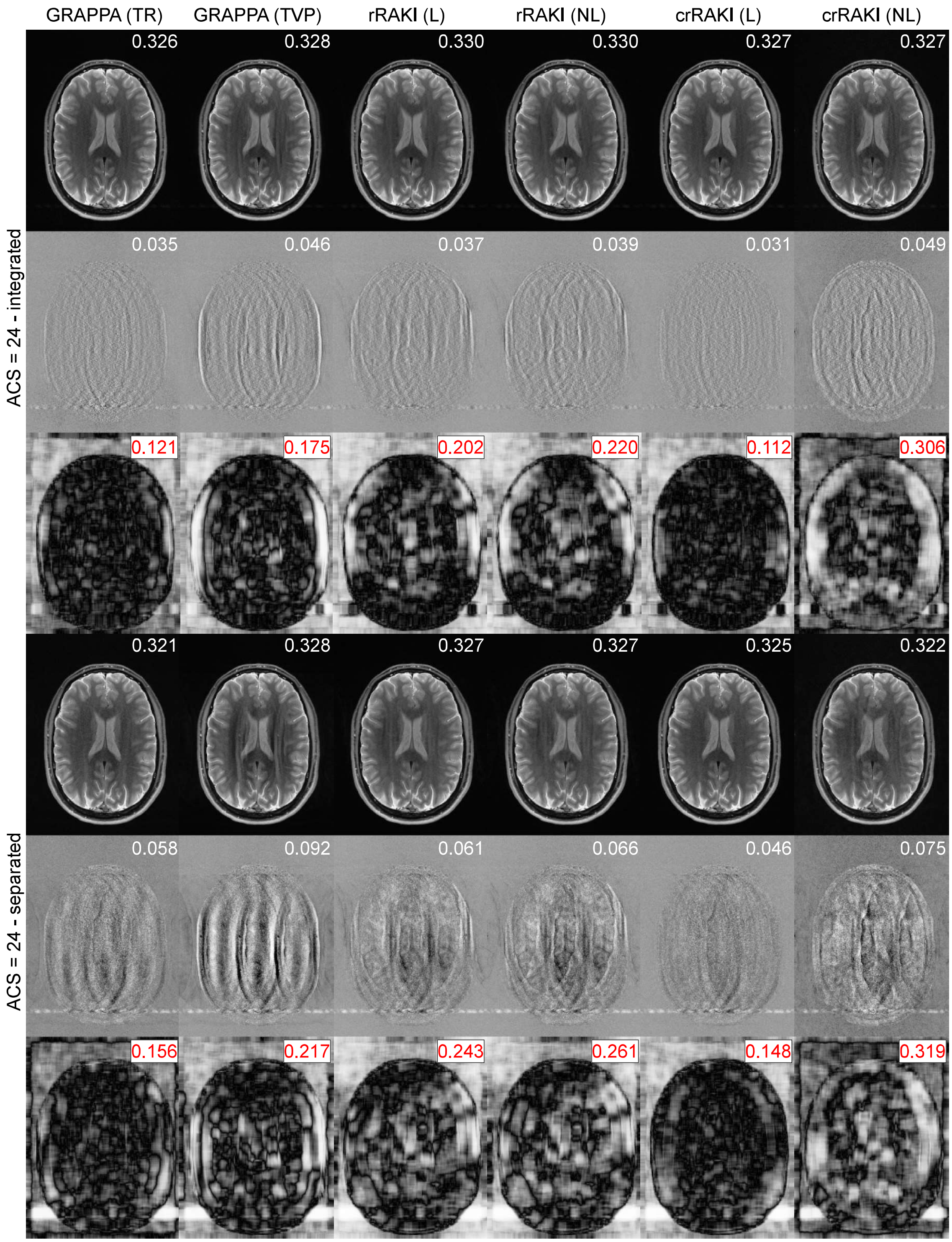

FIGURE S5 Head-to-head comparison of the reconstruction of in-house T2W data for two ACS modes (integrated: top; separated: bottom), R_y=4, ACS size 24, R_{eff}=3.09 and 3.78, respectively). For each ACS mode, the first row if the reconstructed magnitude image, the second row is the residual, the third row is the COBRA map. The numbers on the top-right corners are respectively the Blur metrics, the mean RMSE, the COBRAI taken over a brain mask. This illustrates that incorporating non-linearities, such as in rRAKI, is detrimental in both ACS modes, but particularly in separated ACS mode that requires to estimate more missing lines. The crRAKI Linear method demonstrates superior capability in reconstructing high-quality images with enhanced fidelity and accuracy.

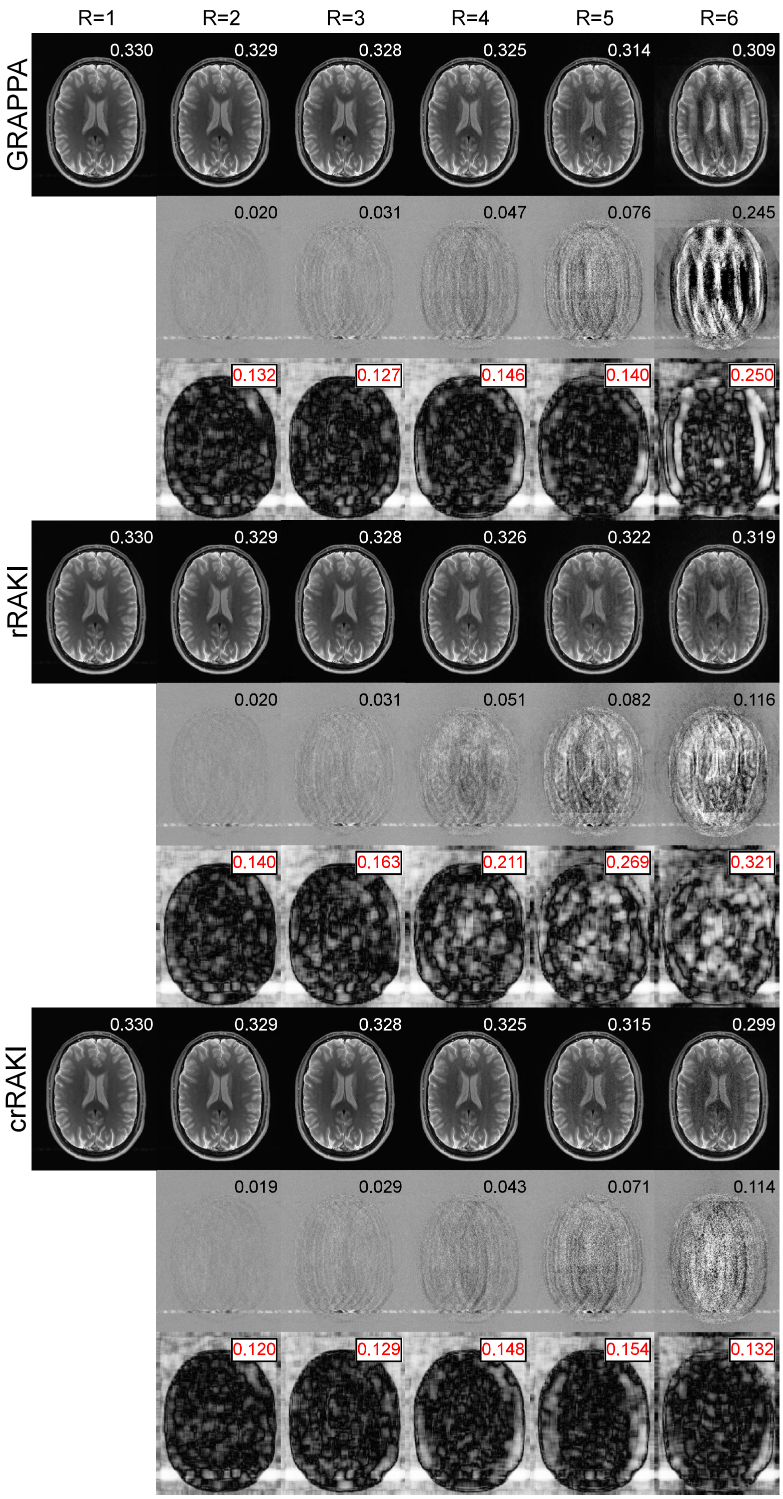

FIGURE S6 Qualitative comparison of GRAPPA, rRAKI, and crRAKI is presented in terms of their ability to reconstruct images at various acceleration rates (R = 2, 3, 4, 5, 6) for N_y^{acs}=40 (R_{eff} = 1.94, 2.82, 3.65, 4.43 and 5.17). Residual maps and fully sampled images are used as the ground truth for this comparison. Residual maps are represented in grayscale, with the brightness of each pixel indicating the magnitude of the absolute difference with the same window level. By examining the residual map, we can assess the quality of images by examining the presence of artifacts and comparing the residual levels. At R_y=4,5 structured artifacts start to appear on GRAPPA and rRAKI reconstructions whereas crRAKI has reduced the number of artifacts. At R_y=6, GRAPPA methods have degraded reconstruction rRAKI has a noisier reconstruction, and crRAKI shows better preservation of the global feature which makes it visually acceptable. The numbers on the top-right corners are respectively the Blur metrics, the mean RMSE, the COBRAI taken over a brain mask.
